# Identification and analysis of CYP450 and UGT supergene family members from the transcriptome of *Aralia elata* (Miq.) seem reveal candidate genes for triterpenoid saponin biosynthesis

**DOI:** 10.1186/s12870-020-02411-6

**Published:** 2020-05-13

**Authors:** Yao Cheng, Hanbing Liu, Xuejiao Tong, Zaimin Liu, Xin Zhang, Dalong Li, Xinmei Jiang, Xihong Yu

**Affiliations:** 1 College of Horticulture and Landscape Architecture, Northeast Agricultural University, Harbin, 150030 Heilongjiang China; 2 Key Laboratory of Biology and Genetic Improvement of Horticulture Crops (Northeast Region), Ministry of Agriculture, Northeast Agricultural University, Harbin, 150030 Heilongjiang China

**Keywords:** *Aralia elata* (Miq.) seem, Cytochrome P450, UGT, Transcriptome-wide identification, Triterpenoid saponin, Subcellular localization

## Abstract

**Background:**

Members of the cytochrome P450 (CYP450) and UDP-glycosyltransferase (UGT) gene superfamily have been shown to play essential roles in regulating secondary metabolite biosynthesis. However, the systematic identification of CYP450s and UGTs has not been reported in *Aralia elata* (Miq.) Seem*,* a highly valued medicinal plant.

**Results:**

In the present study, we conducted the RNA-sequencing (RNA-seq) analysis of the leaves, stems, and roots of *A. elata,* yielding 66,713 total unigenes. Following annotation and KEGG pathway analysis, we were able to identify 64 unigenes related to triterpenoid skeleton biosynthesis, 254 CYP450s and 122 UGTs, respectively. A total of 150 CYP450s and 92 UGTs encoding > 300 amino acid proteins were utilized for phylogenetic and tissue-specific expression analyses. This allowed us to cluster 150 CYP450s into 9 clans and 40 families, and then these CYP450 proteins were further grouped into two primary branches: A-type (53%) and non-A-type (47%). A phylogenetic analysis of 92 UGTs and other plant UGTs led to clustering into 16 groups (A-P). We further assessed the expression patterns of these CYP450 and UGT genes across *A. elata* tissues, with 23 CYP450 and 16 UGT members being selected for qRT-PCR validation, respectively. From these data, we identified CYP716A295 and CYP716A296 as the candidate genes most likely to be associated with oleanolic acid synthesis, while CYP72A763 and CYP72A776 were identified as being the most likely to play roles in hederagenin biosynthesis. We also selected five unigenes as the best candidates for oleanolic acid 3-O-glucosyltransferase. Finally, we assessed the subcellular localization of three CYP450 proteins within *Arabidopsis* protoplasts, highlighting the fact that they localize to the endoplasmic reticulum.

**Conclusions:**

This study presents a systematic analysis of the CYP450 and UGT gene family in *A. elata* and provides a foundation for further functional characterization of these two multigene families.

## Background

*Aralia elata* (Miq.) Seem is a member of the *Araliaceae* family and grows widely throughout Korea, Japan, Russia, and China, where it is used as both a food and a medicinal plant [1]. Owing to their unique taste, young *A. elata* shoots are commonly eaten in many regions of Asia [2]. In addition, the roots and bark of these plants are often incorporated into the traditional Chinese medicine known as “cilaoya”. Previous phytochemical studies have determined that triterpenoid saponins are the primary bioactive substances within *A. elata*, and these compounds have been employed for the treatment of neurasthenia [3], diabetes mellitus [4], hepatitis [5], and gastrospasm [6]. A number of distinct triterpene saponins (chikusetsusaponins Iva and IV and aralosides A, B, V, VII, and X) have been isolated from the leaves [7, 8] and root bark [9, 10]. As such, *A. elata* is ideal for the study of the biosynthesis of triterpenoid saponins, and in particular those of hederagenin and oleanane-types.

**Fig. 1:**
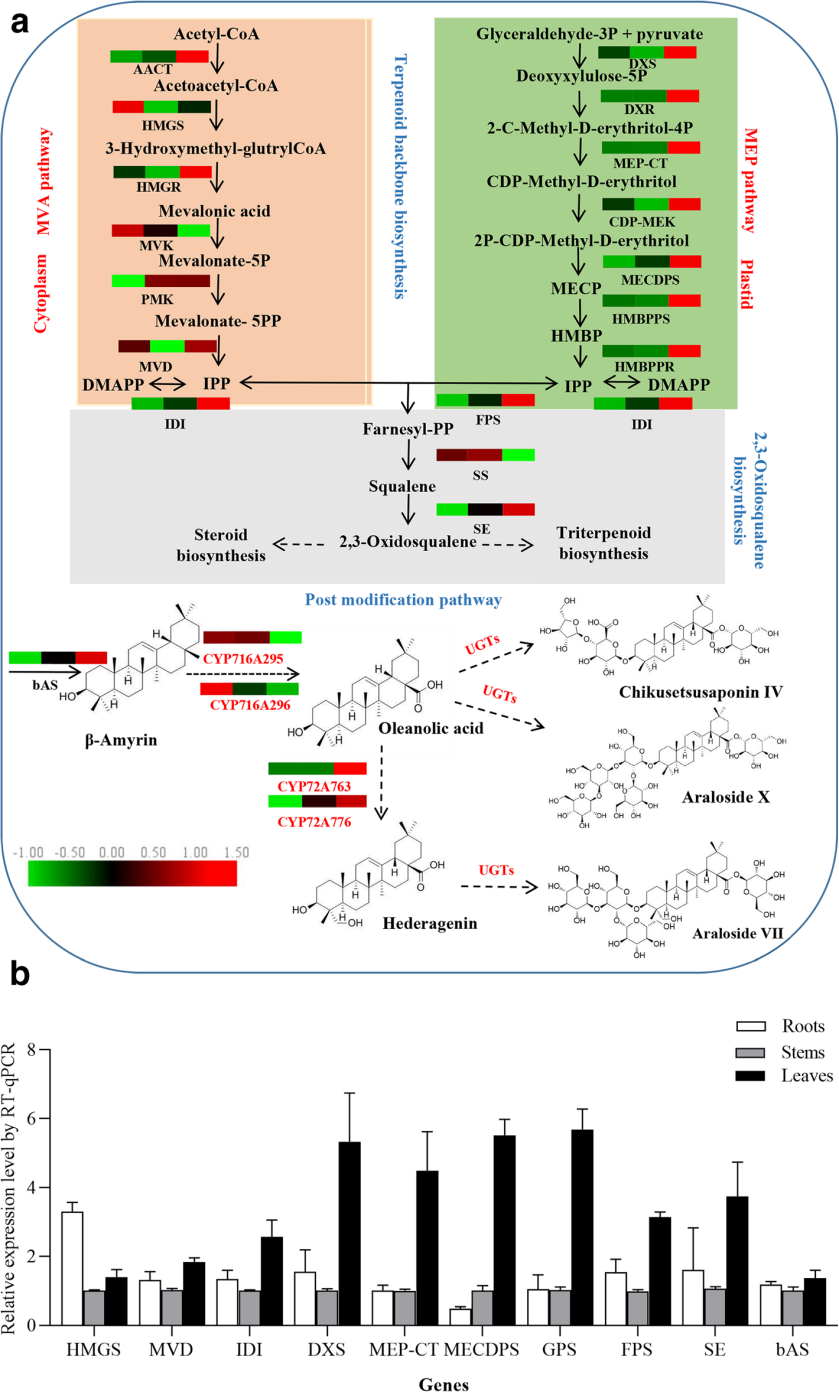
(**a**) Putative pathways of triterpenoid biosynthesis in *A. elata*, with the enzymes identified herein included in this diagram. The heatmap highlights the patterns of expression for these genes in the root, stem, and leaf tissues, with RPKM values used for normalization and color-coding conducted accordingly. Broken arrows indicate putative araloside biosynthesis steps that involve CYP450s and UGTs. (**b**) The selected genes putatively involved in triterpene saponin backbone biosynthesis were quantified via qRT-PCR, with the 2^−△△CT^ approach used to assess gene expression levels relative to those in stem tissues. *GAPDH* was used for normalization, and data are included with standard deviations

Triterpenoids and steroids are a highly diverse group of natural products, and they largely share a metabolic pathway that can be divided into three parts [11] (Fig. 1a). Terpenoids are initially generated from isopentenyl diphosphate (IPP), which derived from the cytosolic mevalonic acid (MVA) or the plastidal methylerythritol phosphate (MEP) pathways. The MVA pathway is responsible for triterpenoid biosynthesis. IPP can also undergo isomeric conversion to DMAPP (dimethylallyl diphosphate) through the action of IDI (isopentenyl diphosphate isomerase) [12]. Farnesyl diphosphate (FPP) synthase (FPS) is responsible for catalyzing IPP and DMAPP units to undergo sequential condensation, along with the reaction intermediate geranyl diphosphate (GPP), yielding FPP. Two FPP molecules are then catalyzed by squalene synthase (SS) and squalene epoxidase (SE), resulting in the formation of 2,3-oxidosqualene. Next, 2,3-oxidosqualene cyclization is driven by oxidosqualene cyclases (OSCs), yielding different triterpenoid backbones [13], including β-amyrin, phytosterol, dammarane and lupane [11]. This step is thus a critical branching point for triterpenoid and phytosterol biosynthesis [14] (Fig. 1a). Finally, CYP450s and UGTs control the subsequent oxidation, hydroxylation, and glycosylation steps to yield triterpenoid saponins and phytosterol [15]. In the context of pentacyclic triterpenoid saponin biosynthesis, CYP450s introducing a carboxyl group at C-28 and hydroxyl groups at C-2β, C-16α, C-23 and C-24 of the β-amyrin skeleton are predicted to form multiple sapogenins, such as oleanolic acid, hederagenin and glycyrrhetinic acid [16]. UGTs that can glycosylate the sapogenins at the C-3 and C-28 positions are predicted to form monodesmosidic or bisdesmosidic saponins with specific structures and activities [17]. Prior studies have highlighted the key roles of different enzymes in the synthesis of the triterpene skeleton, whereas the enzymes involved in the post-biosynthetic diversification of these proteins remain to be fully characterized.

CYP450 and UGT genes in plants are highly diverse and are essential to the diversification of triterpenoid saponin structures [18, 19]. Because there are so many members in these gene families, it has been difficult to fully elucidate their roles in this biosynthetic context [20]. RNA-seq and other sequencing technologies, however, now offer an opportunity to more readily identify CYP450s and UGTs [21]. One prior RNA-seq analysis of three *Panax notoginseng* plants led the authors to identify 350 and 342 predicted unigenes encoding CYP450s and UGTs, respectively [22]. Similarly, a transcriptomic assessment of *Ilex* species allowed researchers to identify 233 CYP450s and 269 UGTs, of which 14 CYP450s and 1 UGT were proposed to play roles in triterpenoid saponin biosynthesis [12]. Even though *A. elata* has great economic and pharmacological utility, there have been no transcriptomic databases for this plant constructed to date, and no studies have systematically identified genes involved araloside biosynthesis.

In this study, we performed RNA-sequencing to analyze the transcriptomic profiles of three different *A. elata* tissues. We further conducted a systematic analysis of *A. elata* CYP450 and UGT family members at the transcriptomic level. Next, based on phylogenetic analyses and the expression profiles, we identified candidate CYP450 and UGT family member genes involved in araloside biosynthesis. Last, three candidate CYP450s were then subjected to subcellular localization analyses. The results of this study will help to foster further research aimed at better understanding the role of CYP450 and UGT genes in the post-biosynthestic modification of triterpenoid saponin biosynthesis in *A. elata*.

## Results

### Quantitative analysis of *A. elata* aralosides

The saponins present within *A. elata* primarily contain oleanolic acid and hederagenin aglycone [3], with significant variations in total and monomer araloside accumulation among tissues in these plants. Specifically, the leaves of *A. elata* have been found to contain the largest quantity of these saponins, with progressively lower levels found in root and stem tissues. The roots of these plants contained higher oleanolic acid levels than did the other tested tissues, whereas hederagenin levels were highest in leaves relative to roots and stems (Table 1; Additional file 1: Figure S1). Two selected oleanane-type saponins (chikusetsusaponin IV and araloside X) and one hederagenin-type saponin (araloside VII) were also detected in these *A. elata* samples, with root chikusetsusaponin IV levels being fairly high, whereas they were minimal in leaf and stem tissues. In contrast, we detected high levels of aralosides VII and X in the leaves of these plants, suggesting that different UGTs were responsible for their generation. These tissue-specific saponin distribution patterns serve as a valueble reference for research efforts aimed at identifying those CYP450s and UGTs involved in araloside production in *A. elata*.

**Table 1:** The aralosides contents in different tissues of *A. elata*

Aralosides contents	Roots	Stems	Leaves
Total saponins (mg·g^− 1^)	37.27 ± 0.70b	6.26 ± 1.23c	52.74 ± 3.40a
Oleanolic acid (μg·g^− 1^)	44.70 ± 5.23a	11.95 ± 1.68b	8.57 ± 1.94b
Hederagenin (μg·g^− 1^)	9.05 ± 0.34c	88.71 ± 12.10b	349.97 ± 24.52a
Chikusetsusaponin IV (μg·g^−1^)	573.89 ± 12.96a	26.18 ± 1.19b	10.181 ± 0.95c
Araloside VII (μg·g^−1^)	ND	ND	97.54 ± 15.08a
Araloside X (μg·g^−1^)	9.63 ± 0.28b	9.09 ± 0.49b	114.99 ± 5.02a

ND not detected. Values are mean ± SD. Different letters within a row indicate significant differences at *P* < 0.05

### De novo *A. elata sequence assembly*

To identify genes pertaining to saponin biosynthesis in these *A. elata* plants, we next employed an Illumina HiSeq 4000 platform to sequence the total RNA transcriptomes in root, leaf, and stem tissue samples. In total, this approach yielded 448,112,618 reads that were assembled into 82,238 contigs, with the longest being 16,016 bp, and with an average contig length of 1058 bp. We were then able to assemble these contigs into 66,713 unigenes with a 1846 bp N50 length (Table 2). Next, these unigenes were annotated with the KEGG, UniProt, NCBI nonredundant nucleotide (Nt), and Nr databases via use of the BLASTN and BLASTX algorithms, leading to the annotation of 35,232 (52.81%) unigenes (Additional file 2: Figure S2). These transcriptome sequence data have been deposited in the NCBI Short Read Archive (SRA) under the accession number SRP216867 and BioProject accession PRJNA555256.

**Table 2:** Summary of the RNA-seq analysis of *A. elata*

Total of raw reads	448,112,618
Total assembled bases	66,367,722,247
GC percentage	38.83
Number of contigs	82,238
Maximum length of contigs (bp)	16,016
Minimum length of contigs (bp)	201
Average length of contigs (bp)	1058
N50 of contigs (bp)	1846
Number of unigenes	66,713

### Enrichment of terpenoid backbone and triterpenoid biosynthetic pathways

After annotating 7291 *A. elata* unigenes using the KEGG database, 79 and 47 unigenes were found to be associated with the terpenoid backbone and sesquiterpenoid/triterpenoid biosynthetic pathways respectively, containing the MVA and MEP pathways as well as the 2,3-oxidosqualene biosynthetic pathway (Fig. 1a). A KEGG pathway analysis indicated that 14 unigenes encoding 6 enzymes (AACT, HMGS, HMGR, MVK, PMVK, and MVD) were associated with the MVA pathway. Of these, 5 unigenes were annotated as HMGR, which is essential in this pathway as it catalyzes the conversion of 3-hydroxymethyl-glutrylCoA into meralonic acid (Fig. 1a). However, in the plastid, IPP is formed through the MEP pathway, which begins with pyruvate and glyceraldehyde-3-phosphate. A total of 22 unigenes encoding 9 enzymes (DXS, DXR, MEP-CT, COP-MEK, MECDPS, HMBPPS, HMBPPR, IDI, and GPS) were annotated as being involved in this pathway (Fig. 1a). Additionally, 28 unigenes encoding 4 putative enzymes (FPS, SS, SE, and bAS) were found to be associated with carbocyclic biosynthesis (Additional file 3: Table S1). For the majority of these genes, we were able to map > 1 unigene to a given gene or gene family (Additional file 3: Table S1), suggesting that these sequences may correspond to multiple copies or partial fragments of a given gene [23]. In each of enzymatic steps, those unigenes that showed high identity with functionally characterized genes and were differentially expressed in different tissues were next selected and arranged into a heat map. As shown in Fig. 1a, significant differential expression of these genes was evident in different *A. elata* tissues. Interestingly, all genes involved in the MEP pathway exhibited more robust expression in leaves relative to roots or stems, whereas genes involved in the MVA pathway other than HMGS, MVK, and SS were also expressed at higher levels in leaves. To confirm that these RNA-seq findings were accurate, 10 of these 19 genes were selected, and their expression profiles in a range of tissues were assessed via qRT-PCR. In agreement with our transcriptomic data, the selected genes were expressed at higher levels in leaves, with the exception of HMGS, which was expressed at higher levels in roots (Fig. 1b). These findings indicate that key triterpenoid skeleton biosynthesis reactions mainly occurred in leaves, explaining why leaves were found to contain higher saponin concentrations (Table 1).

### *A. elata* CYP450 identification and phylogenetic analysis

Through our transcriptomic analysis, we were able to identify 254 CYP450 genes in these *A. elata* samples. This number of total CYP450 unigenes (254) was lower than the number identified in studies of *Arabidopsis thaliana* (272) and *Panax ginseng* (484). To obtain more comprehensive understanding of the CYP450 gene family, we classified 150 CYP450-encoding proteins > 300 amino acids in length via alignment with the CYP450 database using allelic, subfamily, and family variant cutoff values of 97, 55, and 40%, respectively [24]. Prof. David Nelson named these CYP450s according to reference sequences within a carefully annotated CYP450 reference database. We were ultimately able to classify these CYP450s into 9 clades, 40 families and 75 subfamilies, with 53% being A-type CYP450s and 47% being non-A-type CYP450s (Additional file 4: Table S2). The CYP71 clan represented all A-type CYP450s, containing 79 genes belonging to 17 families (CYP71, CYP73, CYP75-CYP78, CYP80-CYP82, CYP84, CYP89, CYP92, CYP98, CYP701, CYP706, CYP712 and CYP736).

**Fig. 2:**
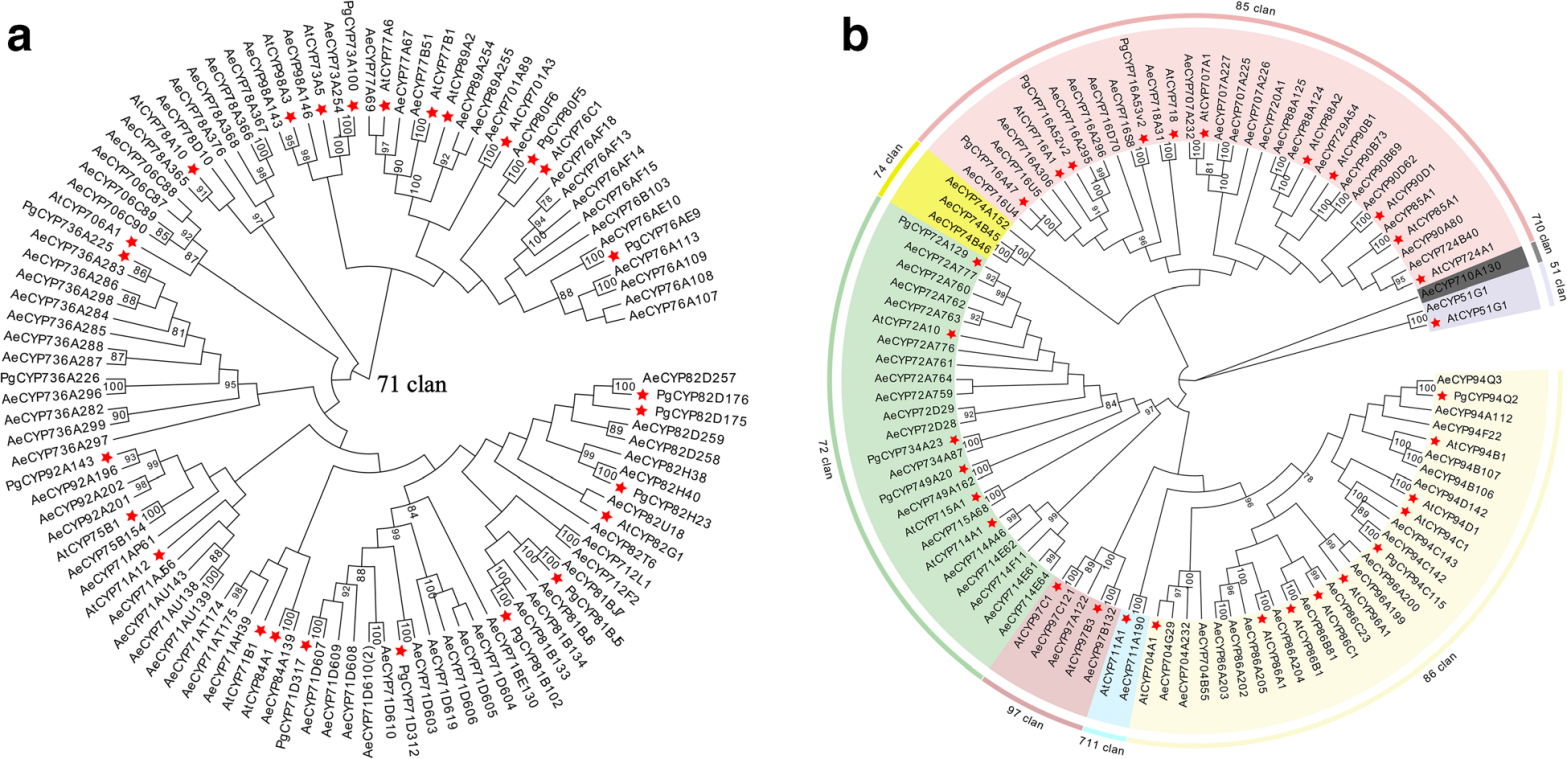
(**a**) Phylogenetic tree of A-type CYP450s from *A. elata* (Ae) and *Arabidopsis* (At) and *Panax ginseng* (Pg). The representative CYP450 family members from *Arabidopsis and P. ginseng* are marked with red stars. (**b**) Phylogenetic tree of non-A-type CYP450s from *A. elata* (Ae) and *Arabidopsis* (At) and *Panax ginseng* (Pg). Representative CYP450s from *Arabidopsis and P. ginseng* are indicated with red stars

To further explore the functional roles and evolutionary relationships for these *A. elata* CYP450s, 150 CYP540s along with 57 CYP450s from *A. thaliana* (37) and *P. ginseng* (20) were used to generate NJ phylogenetic trees for A-type (Fig. 2a) and non-A-type (Fig. 2b) CYP450s, separately. As shown in Fig. 2a, the 79 CYP71 members were assigned to 17 families forming a single clade along with 28 representative CYP450s. The phylogenetic tree for non-A-type CYP450s separated them into 8 clades, including three single-family clans (CYP51, CYP710 and CYP711) and five multifamily clans (CYP72, CYP74, CYP85–86 and CYP97), CYP85, CYP86, and CYP72 were the largest three clans, containing 23, 20, and 17 CYP450s, respectively, while CYP51, CYP710, and CYP711 each contained only a single member (Fig. 2b).

### *A. elata* UGT identification and phylogenetic analysis

We annotated 122 unigenes in the *A. elata* transcriptome as UGTs encoding proteins that were 77–704 amino acids in length. We then omitted those unigenes that encoded proteins shorter than 300 amino acids, yielding 92 UGTs that underwent blastp with those of functionally characterized plant UGTs, with those having > 40% protein sequence similarity being incorporated into the same family (Additional file 5: Table S3). We separated 87 UGTs into 22 UGT families, while 5 UGTs (Unigene0019368; Unigene0025405; Unigene0034502; Unigene0034503; Unigene0060195) had deduced amino acid sequences < 40% identical to representative sequences and thus could not be assigned. The UGT73 family was the largest family, with 16 genes, with the next largest being the UGT 85 family, with 13 members.

**Fig. 3:**
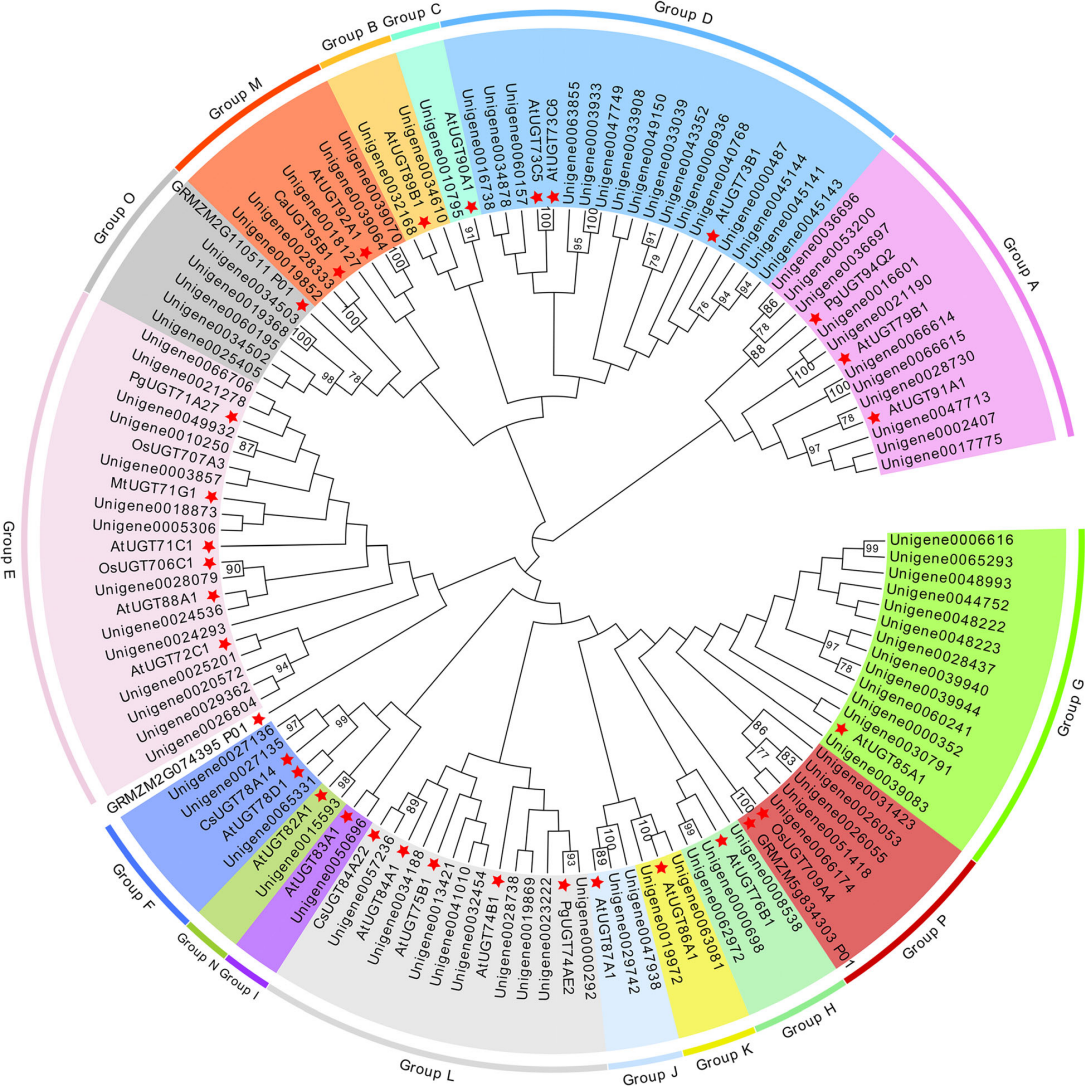
*A. elata* and other plant UGTs were used to prepare a phylogenetic tree, with representative UGT family members indicated with red stars

We aligned *A. elata* UGT amino acid sequences with those of other functionally characterized plant UGTs from *Arabidopsis*, *Panax ginseng*, *Medicago truncatula*, *Oryza sativa*, *Zea mays*, *Cicer arietinum,* and *Crocus sativus* (Additional file 6: Table S4) to construct a phylogenetic tree. These *A. elata* UGTs were phylogenetically separated into 16 groups, including 14 conserved groups (A-N) that were identified in *Arabidopsis* and two novel groups (O and P; Fig. 3) identified in maize [25, 26]. No *A. elata* UGTs were incorporated into group Q. There were 16 UGT73 family members in group D, which was the largest *A. elata* UGT group, while groups C, I and N contained only one UGT each.

### *A. elata* CYP450 and UGT gene tissue-specific expression patterns

**Fig. 4:**
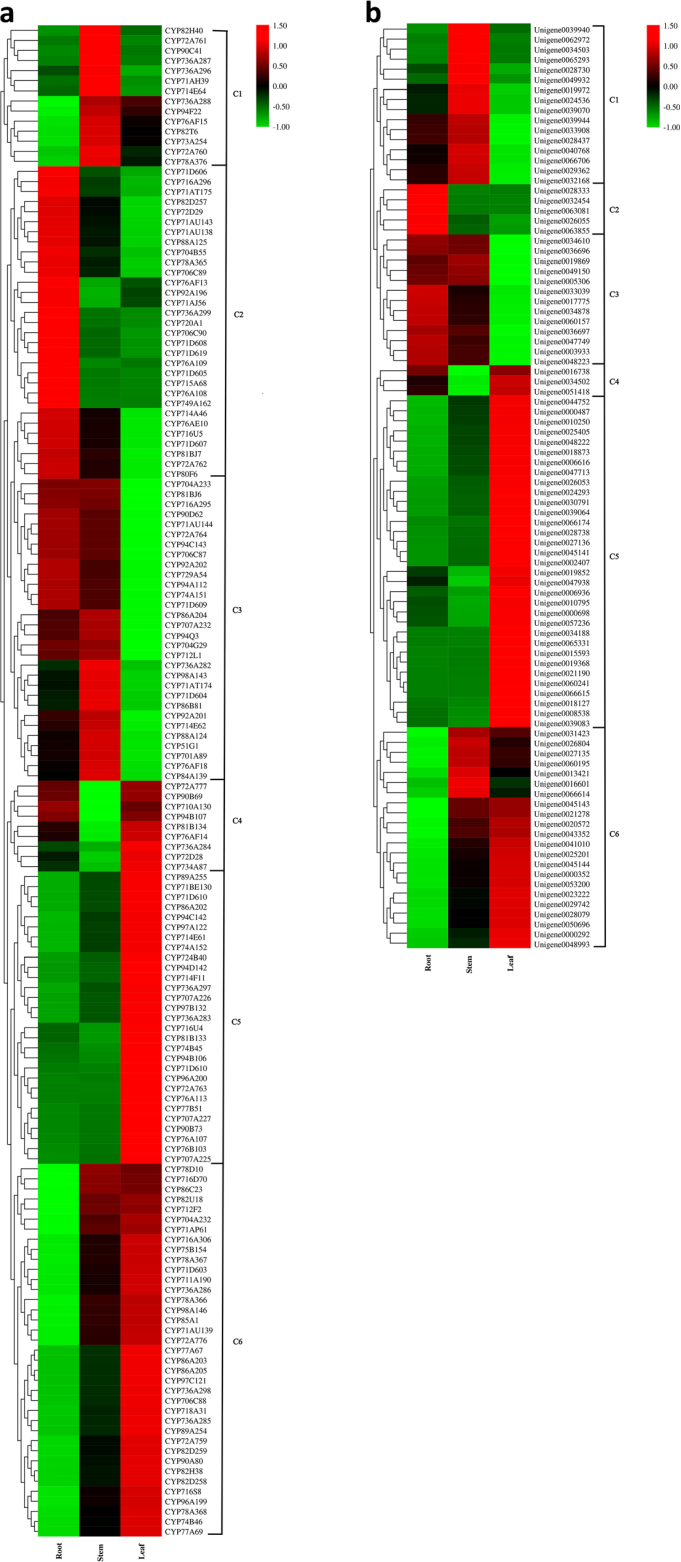
(**a**) *A. elata* CYP450 expression profiles. Hierarchical clustering for these 150 CYP450s was conducted based upon RNA-seq results. (**b**) *A. elata* UGT expression profiles. Hierarchical clustering for these 92 UGTs was performed based upon RNA-seq results

To understand the tissue-specific expression of CYP450s and UGTs in *A. elata*, we next conducted an analysis of their expression profiles in the three different tissues that had been subjected to RNA-seq analysis. Of these 150 CYP450s and 92 UGTs, we found that 132 (87.42%) and 78 (84.78%) exhibited patterns of differential expression across these tissues, respectively. A hierarchical clustering analysis was used to assess CYP450 and UGT co-expression patterns in these different analyses, with an expression profile heat map for these genes being constructed according to their RPKM normalized expression values. This clustering analysis led to the assignment of 150 CYP450s and 92 UGTs to six clusters (C1-C6; Fig. 4a and b). The CYP450s that were most highly expressed in roots (31 genes), leaves (29 genes), and stems (15 genes) were grouped into clusters C2, C5, and C1, respectively. In addition, those CYP450s in clusters C3 (31 genes), C4 (8 genes), and C6 (36 genes) were expressed at the lowest levels in leaf, stem, and root tissues, respectively. An assessment of different *A. elata* tissues indicated that CYP450s were expressed at high levels in leaves (48.67%) and roots (41.33%) (Fig. 4a). However, a high proportion (63.04%) of these UGTs were expressed at high levels in leaves relative to roots (19.57%) and stems (17.39%) (Fig. 4b). This suggests that the modification of UGTs primarily occurs in leaves, explaining the greater variety of secondary metabolites in leaves.

**Fig. 5:**
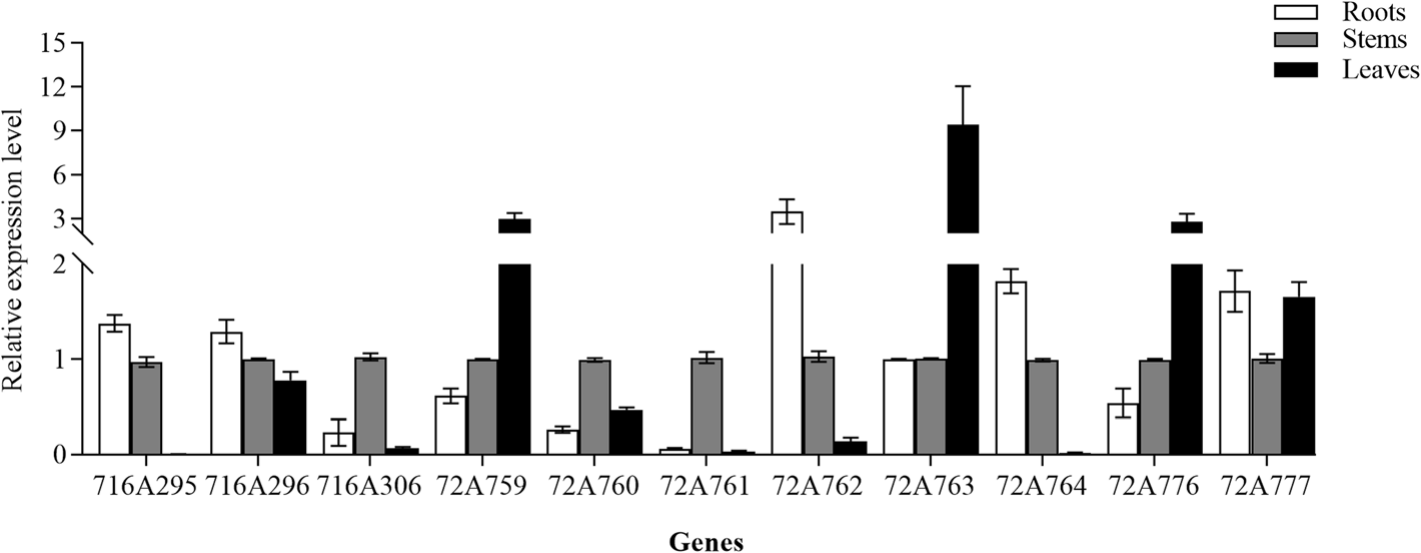
Comparison of the expression levels of 3 CYP716A and 8 CYP72A genes in different tissues

A qRT-PCR approach was further used to validate these transcriptomic findings, with the expression of 12 randomly selected CYP450s being quantified (Additional file 7: Figure S3). These CYP450 expression profiles were consistent with the RPKM values, confirming the validity of our RNA-seq data. Previous research has shown that members of the CYP72A and CYP716A subfamilies are the primary CYP450s involved in pentacyclic triterpenoid saponin biosynthesis (Additional file 8: Table S5). As such, we next specifically focused on the expression patterns of the 3 CYP716A and 8 CYP72A genes identified in the *A. elata* transcriptome. The qRT-PCR profiles for these genes revealed that two CYP716A genes (CYP716A295 and CYP716A296) and two CYP72A genes (CYP72A762 and CYP72A764) were expressed at high levels in root tissues relative to stems and leaves. Expression of these four genes was consistent with measured oleanolic acid contents. In contrast, CYP72A759, CYP72A763, and CYP72A776 were expressed at higher levels in leaves and at lower levels in stems and roots, and the expression patterns of these three genes were consistent with hederagenin contents (Fig. 5). These results provided a reference for the selection of CYP450 candidates related to triterpenoid saponin biosynthesis.

**Fig. 6:**
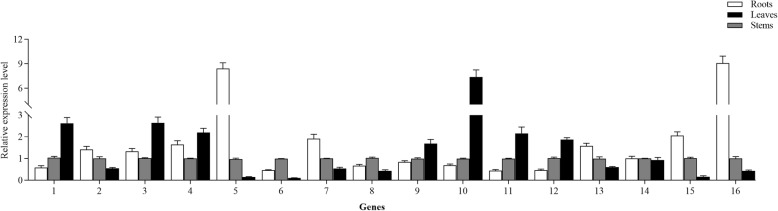
Comparison of the tissue-specific expression levels of 16 UGT73 family members. Numbers 1–16 correspond to Unigene0000487, Unigene0003933, Unigene0006936, Unigene0016738, Unigene0033039, Unigene0033908, Unigene0034878, Unigene0040768, Unigene0043352, Unigene0045141, Unigene0045143, Unigene0045144, Unigene0047749, Unigene0049150, Unigene0060157, and Unigene0063855

Considering that the members of the UGT73 family primarily catalyzed glycosylation of oleanolic acid and hederagenin at the C-3 and C-28 sites (Additional file 9: Table S6), the tissue-specific expression profiles of 16 UGT73 members identified in this study were analyzed via qRT-PCR. As shown in Fig. 6, 15 out of these 16 UGT73 members exhibited significant differential expression profiles in three tissues. Of these, 7 UGTs (Unigene0000487, Unigene0006936, Unigene0016738, Unigene0043352, Unigene0045141, Unigene0045143, and Unigene0045144) were most highly expressed in leaves, while six UGTs (Unigene0003933, Unigene0033039, Unigene0034878, Unigene0047749, Unigene0060157, and Unigene0063855) were mostly expressed in roots, with progressively lower levels in stems and leaves. These findings indicate that most UGT73 members were highly expressed in *A. elata* roots and leaves.

### Identification of candidate CYP450s involved in triterpenoid biosynthesis

**Fig. 7:**
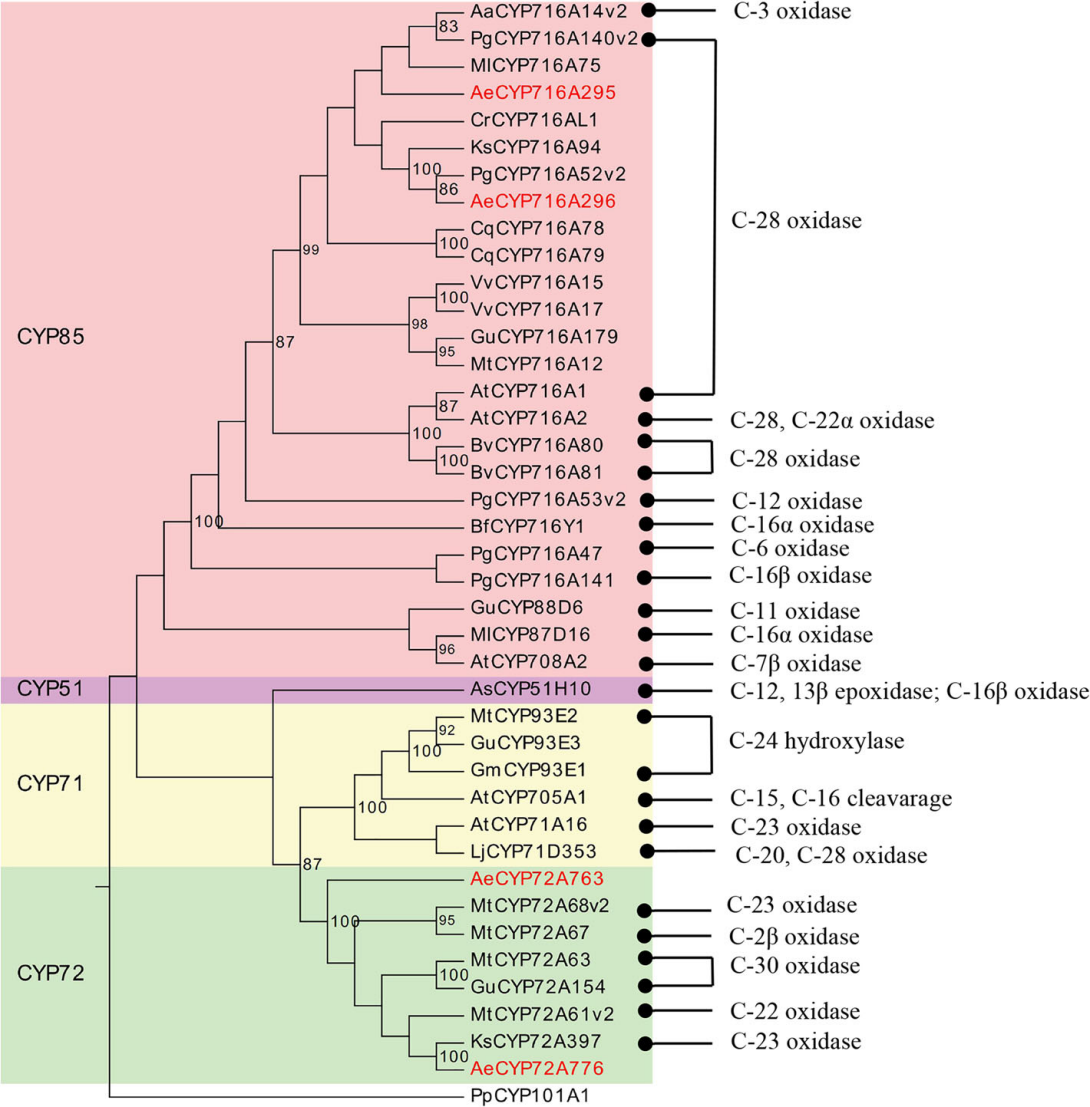
Phylogenetic tree of previously characterized triterpenoid biosynthesis CYP450s and those *A. elata* CYP450s isolated in this study (in red). The known biochemical activities of these P450s are indicated on the right. CYP101A1 from *Pseudomonas putida* (accession No. 2 L8 M_A) was used as an outgroup in the phylogenetic tree

**Fig. 8:**
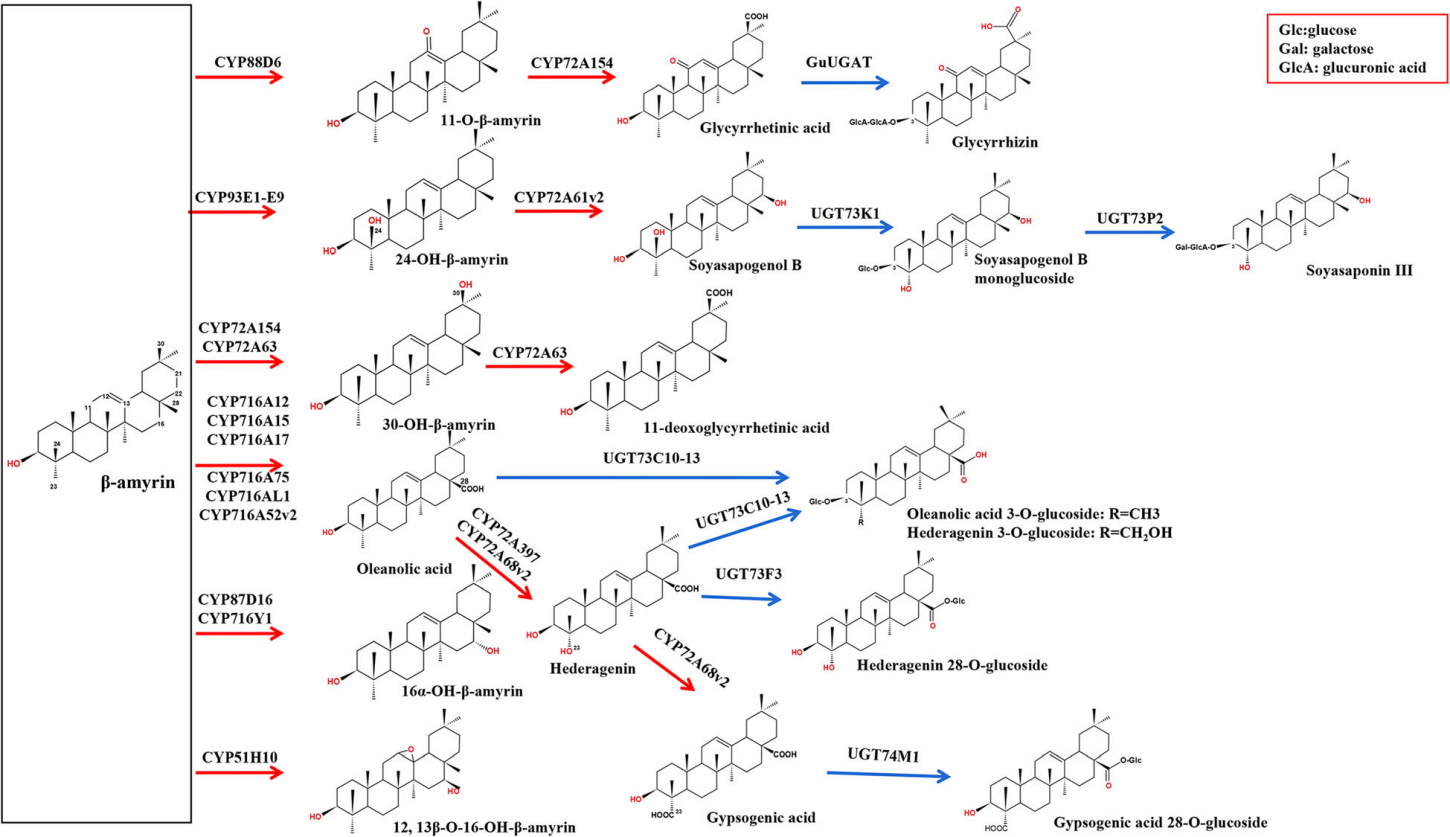
Modification of pentacyclic triterpenoid saponins catalyzed by characterized CYP450s and UGTs. CYP450-catalyzed steps are indicated with red arrows, UGT-catalyzed steps are indicated with blue arrows, and the different types of sugar moieties attached by UGTs are shown in the red box

Hederagenin aglycone and oleanolic acid are the major sapogenins in *A. elata,* and as such, we specifically assessed CYP450s related to the synthesis of these sapogenins*.* To date, a total of 36 CYP450s have been found to play roles in triterpenoid biosynthesis (Fig. 7; Additional file 8: Table S5). The CYP716A and CYP72A subfamilies are the primary CYP450 gene families involved in pentacyclic triterpenoid saponin diversification, with the CYP716A family being the largest multifunctional C28-oxidase family involved in such oleanane-type triterpenoid saponins biosynthesis [16, 27, 28] (Fig. 8). We were able to identify 3 CYP716A genes (CYP716A295, CYP716A296, and CYP716A306) and 8 CYP72A genes (CYP72A759–764, CYP72A776–777) in the *A. elata* transcriptome. To identify the most relevant unigenes involved in pentacyclic triterpenoid saponin biosynthesis for further analysis, we conducted BLASTx searches that compared these *A. elata* CYP450s to those 36 CYP450s known to be involved in triterpenoid biosynthesis. This analysis revealed that the *A. elata* CYP716A295 and CYP716A296 exhibited 93.97 and 94.39% sequence identity with *P. ginseng* CYP716A52v2, respectively, which is a β-amyrin 28-oxidase enzyme involved in oleanolic acid production [29] (Fig. 8). Moreover, CYP716A295 and CYP716A296 were expressed at higher levels in roots relative to stems and leaves, in line with oleanolic acid contents (Fig. 5, Table 1). As such, we selected CYP716A295 and CYP716A296 as the best candidate CYP450s likely to be involved in oleanolic acid biosynthesis in *A. elata.* Two CYP450s (CYP72A397 and CYP72A68v2) have thus far been identified as oleanolic acid 23-oxidases, catalyzing oleanolic acid oxidation into hederagenin [30, 31] (Fig. 8). In the present study, we observed higher expression levels of CYP72A759, CYP72A763, and CYP72A776 in leaf tissues, with progressively lower levels in stems and roots, consistent with the observed hederagenin distribution profile (Fig. 6). A BLASTx analysis further indicated that CYP72A763 and CYP72A776 encoded proteins with 54.92 and 70% identity to the oleanolic acid 23-hydroxylase CYP72A397, respectively. Given these results, we further selected CYP72A763 and CYP72A776 as the CYP450s most likely to be involved in hederagenin biosynthesis, although further functional validation will be necessary.

Phylogenetic analyses revealed CYP716A295 and CYP716A296 to be grouped in the CYP716A subfamily and to be most closely related to *Maesa lanceolata* CYP716A75 and *P. ginseng* CYP716A52v2, respectively, both of which encode β-amyrin 28-oxidase enzymes involved in oleanolic acid production [29, 32]. CYP72A763 and CYP72A776 clustered in the CYP72A group and were most closely related to *M. truncatula* CYP72A68v2 and *Kalopanax septemlobus* CYP72A397, respectively, both of which encode β-amyrin 23-hydroxylase enzymes [30, 31] (Fig. 7).

### Identification of candidate UGTs involved in triterpenoid biosynthesis

**Fig. 9:**
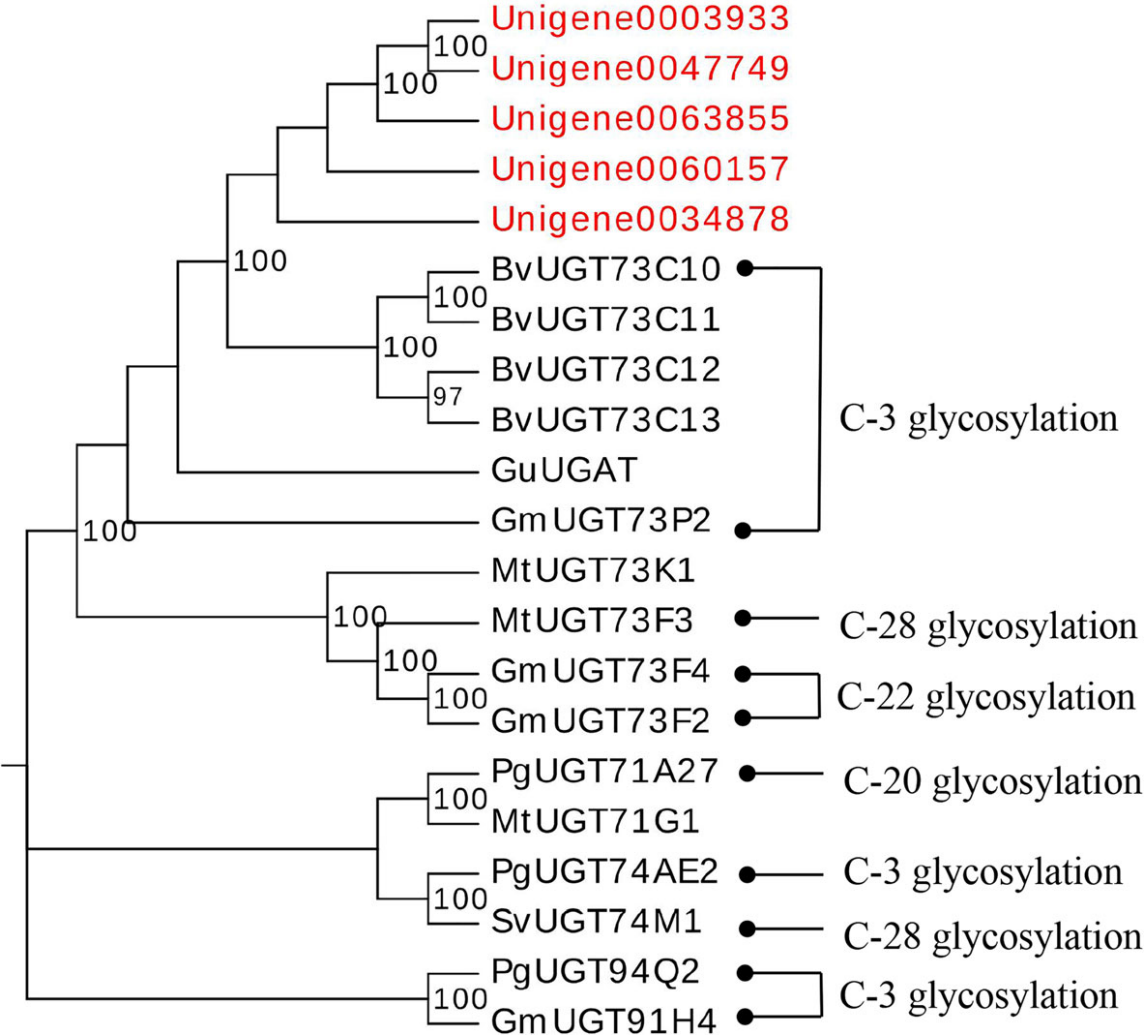
Phylogenetic tree of *A. elata* UGTs identified in the present study (marked with red) and UGTs previously shown to play a role in triterpenoid biosynthesis. Known glycosylation sites for these UGTs are as shown on the right. Glycosylation sites targeted by UGT73K1 and UGT71G1 remain to be determined

To determine which UGTs were involved in araloside glycosylation, blastp was used to compare 92 *A. elata* UGTs to 16 functionally characterized UGTs. We determined that five unigenes (Unigene0003933, Unigene0034878, Unigene0047749, Unigene0060157, and Unigene0063855) exhibited 50–60% identity to *Barbarea vulgaris* UGT73C10–13, which catalyzed the 3-O-glucosylation of oleanolic acid and hederagenin [17] (Fig. 8). This suggests that these unigenes may encode enzymes important for catalyzing oleanolic acid and hederagenin glucosylation at the C-3 position. We also used qRT-PCR to confirm that these five UGTs were expressed at high levels in roots, with lower expression levels in stems and leaves, consistent with observed oleanolic acid distributions in these tissues. This suggests that the 3-O-glucosylation of oleanolic acid occurs in roots. Phylogenetic analyses suggested that these five UGTs were grouped in the UGT73 family and were most closely related to *Barbarea vulgaris* UGT73C10 (Fig. 9). We therefore identified these UGTs as candidates involved in the glycosylation of oleanolic acid at the C-3 position, although future functional assays will be necessary to confirm this result.

### Assessment of the subcellular localization of three CYP450:GFP fusion proteins

**Fig. 10:**
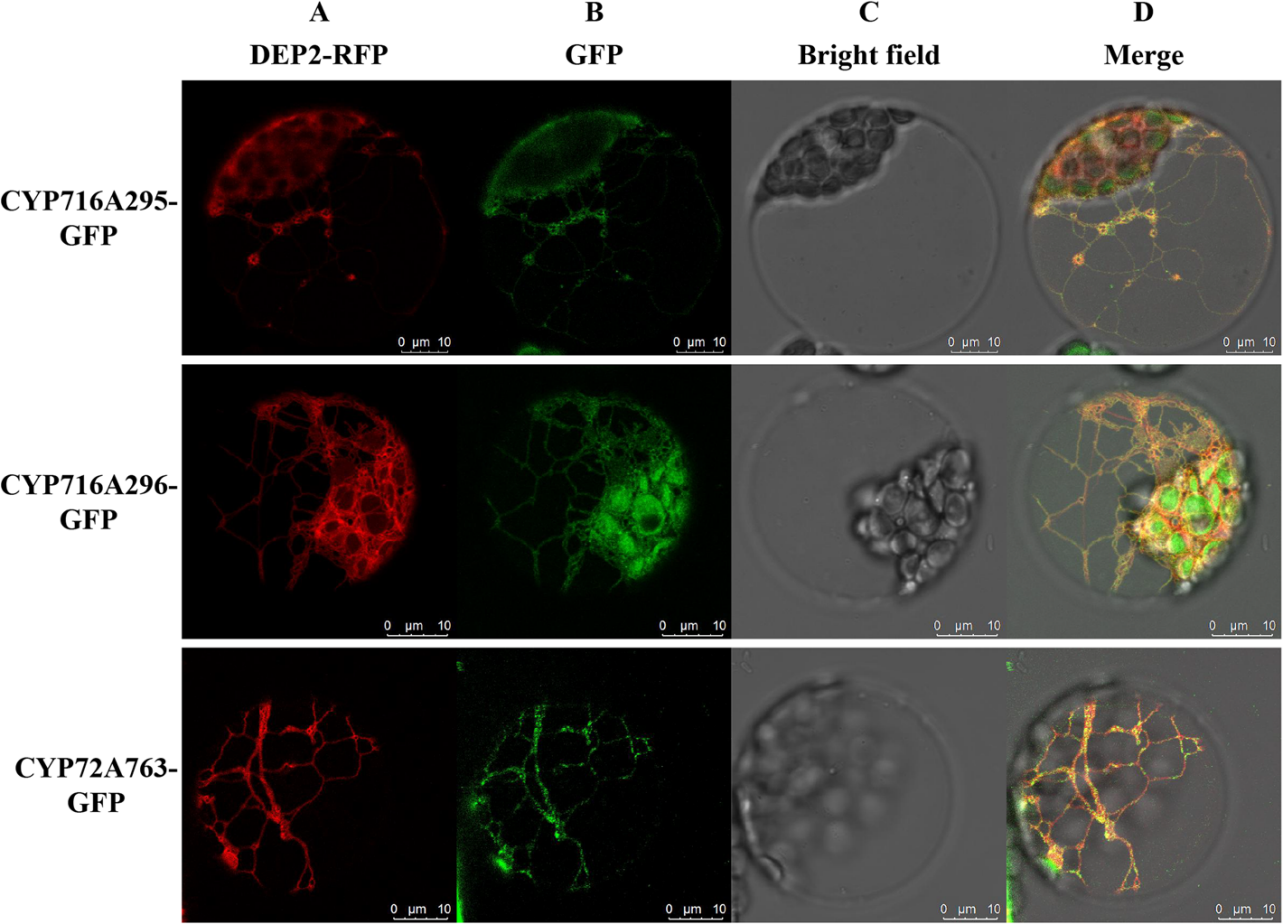
CYP716A295, CYP716A296, and CYP72A763 subcellular localization in *Arabidopsis* protoplasts cotransformed with CYP450s-GFP and the ER marker DEP2-RFP, as analyzed via confocal microscopy. (**a**) The ER is marked by red fluorescence; (**b**) CYP450s are indicated by green fluorescence; (**c**) Bright field illumination is shown in white; (**d**) A merged image of (A, B, C) indicates that these CYP450s are localized to the ER

Almost all CYP450s are membrane-associated proteins that localize to the ER, with relatively few localizing to chloroplasts and mitochondria [33]. As detailed above, all 150 of the *A. elata* CYP450s identified in this study were predicted to localize to the ER. To confirm this prediction, we conducted PEG-mediated transient expression of *Arabidopsis* protoplasts co-transformed with CYP450-GFP reporter proteins and DEP2-RFP (a ER-targeted marker) [34], with CYP716A295, CYP716A296, and CYP72A763 all being selected for this subcellular localization analysis. Consistent with what was observed in *Arabidopsis* protoplasts expressing DEP2-RFP, we found CYP450-GFP proteins to appear as a reticular ribbon upon microscopic examination with overlap between the CYP716A295, CYP716A296, and CYP72A763 GFP fluorescent proteins and DEP2-RFP fluorescence proteins. We therefore concluded that CYP716A295, CYP716A296, and CYP72A763 were enzymes bound to the ER membrane, consistent with the predictions made by Cell-PLoc (Fig. 10).

## Discussion

While both *A. elata* and *P. ginseng* are triterpenoid saponin-rich members of the *Araliaceae* family that are commonly used in traditional medicinal contexts, *P. ginseng* has been far better studied to date, with its genome having been released in the Ginseng Genome Database (http://ginsengdb.snu.ac.kr/). In contrast, there have been minimal molecular biology studies conducted to date focusing on *A. elata*, with no corresponding genomic or transcriptomic data being available for this species in the NCBI database. The present study was the first to conduct a de novo transcriptome analysis of *A. elata*, analyzing three replicates each of root, stem, and leaf tissue samples from these plants. An Illumina HiSeq 4000 platform was used to sequence the libraries prepared from these 9 samples, yielding 66,713 unigenes, of which over half were well-annotated within public databases. The N50 and average lengths of the unigenes were consistent with the effective and high-quality assembly of these sequencing results [35]. The results of this deep sequencing analysis have immense value as a means of identifying those genes involved in the biosynthesis of pharmacologically relevant secondary metabolites in *A. elata*, as evidenced by our identification of CYP450s and UGTs involved in triterpenoid saponins in these plants.

After the assembly and annotation of the *A. elata* transcriptome in the present study, we were able to begin examining the terpenoid backbone biosynthesis and sesquiterpenoid and triterpenoid biosynthesis pathways in these samples, leading to the identification of 19 functional genes. While these two pathways are well-known to be important for the biosynthesis of terpenoids and sterols in *P. notoginseng* [22] and *Hedera helix* L. [23], the results of the present analysis are the first such comprehensive analysis of these pathways in *A. elata*. Triterpenoids and sesquiterpenoids are synthesized via the MVA pathway, which takes place primarily in the cytoplasm, whereas monoterpenoids, diterpenoids, and tetraterpenoids are synthesized via the MEP pathway, primarily within plastids [11]. Gene expression levels were compared based upon RPKM values, with qRT-PCR being used for verification. These analyses of *A. elata* indicated that the expression levels of genes involved in saponin precursor and skeleton synthesis, and particularly MEP pathway-related genes, were highest in leaves. Similar gene expression patterns were also found in *Panax zingiberensis* and *Cymbopogon winterianus* [36, 37]. We therefore speculated that saponin backbone biosynthesis primarily occurs in leaves. This fact may also explain why we observed a higher abundance of triterpenoid saponins in the leaves of *A. elata* relative to the root and stem tissues.

CYP450s constitute one of the largest enzymatic families, catalyzing irreversible oxidation reactions and being subject to complex functional classification [38]. Over 5100 CYP450 sequences have been identified to date (http://drnelson.uthsc.edu/CytochromeP450.html), with hundreds of these proteins being encoded in the genome of a given plant. For example, 272 functional CYP450s have been identified in *Arabidopsis*, while 355 have been identified in rice [39], and 484 have been identified in *P. ginseng*. In the present study, we conducted systematic identification, nomenclature assignments, and tissue-specific expression analyses of CYP450 genes in *A. elata*. In total, we identified 254 CYP450 genes, and all were novel and had not previously been reported in *A. elata*. We further classified the 150 CYP450s encoding > 300 amino acids into 9 clans, with the CYP727 and CYP746 clans not being identified in the present analysis owing to the absence of an available whole-genome sequence for *A. elata.* The CYP51 clan member genes are thought to be the evolutionarily oldest CYP450s, having evolved from a sterol-metabolizing CYP51 ancestor [39]. We identified only a single CYP51 clan member in the present analysis (CYP51G1). The CYP71 family is the largest CYP450 clan, being composed of 17 different families and making up the entirety of A-type CYP450 genes, playing multiple roles in the biosynthesis of secondary metabolites or natural products [40].

Ever since CYP716A12 was first described as a triterpenoid-oxidizing enzyme that catalyzes α-amyrin, β-amyrin, and lupeol at the C-28 position to ursolic acid, oleanolic acid, and betulinic acid, respectively [28, 41], several other members of this CYP716 family have also been characterized and found to play complex roles in different plants. In the context of pentacyclic triterpenoid synthesis, CYP716 family enzymes have been shown to exhibit oxidation activity at the C-28, C-22α, C-3, and C-16β positions, respectively (Fig. 8). In dammarane-type triterpenoid synthesis, CYP716 family enzymes also exhibit catalytic activity at the C-12 and C-6 positions (Fig. 7). In *A. elata,* the primary saponins are oleanane-type pentacyclic triterpenoids, which are catalyzed by CYP450 genes from β-amyrin at the C-28 position [3] (Fig. 8). This CYP716A subfamily is closely associated with oleanane-type triterpene biosynthesis in several plant species [15, 42]. In the present study, we were able to identify three CYP716A family members (CYP716A295, CYP716A296, and CYP716A306), and we further found that the expression levels of CYP716A295 and CYP716A296 were consistent with oleanolic acid contents. We therefore postulated that CYP716A295 and CYP716A296 most likely to be genes involved in the synthesis of oleanolic acid in *A. elata*.

We also detected significant levels of the hederagenin aglycone sapogenin in *A. elata*, with this compound being produced via the C-23 oxidation of oleanolic acid (Fig. 8). Members of the CYP72A subfamily have been shown to be involved in a variety of sapogenin biosynthesis reactions, with four *M. truncatula* CYP450 genes (CYP72A63, CYP72A61v2, CYP72A67, and CYP72A68v2), one *Glycyrrhiza uralensis* CYP450 gene (CYP72A154), and one *K. septemlobus* gene (CYP72A397) having been shown to be involved in sapogenin biosynthesis [30, 31, 43, 44]. The CYP72A68v2 enzyme in *M. truncatula* has been shown to catalyze the oleanolic acid to gypsogenic acid conversion via intermediate hederagenin formation, while CYP72A397 in *K. septemlobus* produces hederagenin as a single compound [30, 31] (Fig. 8). These CYP450s were proposed to have hydroxylation activity at the C-23 position of the enzyme on the oleanolic acid substrate. A BLASTx analysis suggested that the *A. elata* CYP72A763 and CYP72A776 amino acid sequences shared 54.91 and 70% sequence identity with *K. septemlobus* CYP72A397, respectively. Given that their expression patterns aligned well with the tissue-specific distribution of hederagenin in *A. elata*, we therefore identified CYP72A763 and CYP72A776 as candidate genes involved in hederagenin biosynthesis.

Glycosyl modifications can significantly increase the diversity of plant phytochemicals, with UGT superfamily members catalyzing the glycosylation of these compounds [36]. To date, UGTs have been systematically identified in only certain model plants and industrial crops, including *A. thaliana*, *Brassica* and maize [25, 26, 45]. These past studies have identified UGTs via genome-wide analyses, whereas fewer studies have done so via transcriptome-wide analyses. In the present study, 122 UGT genes were identified in the *A. elata* transcriptome*,* including 92 UGTs encoding > 300 amino acids that were clustered in 16 groups, with 14 highly conserved (A-N) and two novel (O and P) groups identified in maize. This classification scheme is in line with recent findings in peach and *Brassica* species [45, 46]. In addition, we found only one group N UGT in *A. elata* and no group Q members. In contrast to prior findings, group D was the largest group of *A. elata* UGTs, suggesting unique evolutionary specificity in *A. elata*.

Since Achnine et al. [47] first characterized the functions of UGT73K1 and UGT71G1 related to triterpenoid saponin biosynthesis in *M. truncatula*, 14 UGTs have been examined with respect to their biochemical functions (Additional file 9: Table S6). Of these UGTs, the UGT73 family primarily catalyzes the glycosylation of oleanolic acid and hederagenin at the C-3 and C-28 positions. A blast search revealed these five *A. elata* UGTs in the UGT73 family to be closely related to *B. vulgarisa* UGT73C10, which encoded oleanolic acid or hederagenin 3-O-glucosyltransferase [17]. These unigenes were expressed at the highest levels in root tissues, consistent with measured oleanolic acid contents. We therefore hypothesized that these five unigenes were candidate oleanolic acid 3-O-glucosyltransferase genes.

## Conclusion

In this study, leaf, root, and stem transcriptomes from *A. elata* were sequenced for the first time. The resultant large dataset of transcripts and unigenes provided a robust genetic basis for identifying important genes and secondary metabolic pathways in these plants. Based on these transcriptomic data and available databases, two pathways and 19 putative genes related to triterpenoid saponin biosynthesis were discovered. We systematically identified CYP450 and UGT superfamily genes, identifying 254 CYP450s and 122 UGTs for the first time in *A. elata*. Analyses of 150 CYP450 and 92 UGT genes encoding sequences greater than 300 amino acids with respect to their phylogeny and expression patterns in different tissues were further conducted. Through sequence homology analyses, aralosides distribution, and tissue-specific gene expression profiles, four candidate CYP450s and five UGTs related to triterpenoid saponin biosynthesis were identified. Finally, the subcellular localizations of three CYP450 candidates were analyzed. Together, this study provides comprehensive insight into the CYP450 and UGT gene families in *A. elata* and will aid in determining these two gene families’ functions in this and related species.

## Methods

### Plant materials

An *A. elata* cultivar (Plant Materials No. CH02–1-03 in Heilongjiang Crop Committee) was used in this study, which was provided by Prof. Hengtian Zhao (Northeast Institute of Geography and Agroecology, Chinese Academy of Sciences) and was grown in the Wild Plant Germplasm Resources Nursery of Northeast Agricultural University (Harbin, Heilongjiang, China; 45^。^44′21″N, 126^°^43′22″E). Two-year-old plants were used for this study, with samples of roots, leaves, and stems from three biological replicates of these plants being collected, snap frozen, and stored at − 80 °C.

### Saponin content quantification

A slightly modified version of the vanillin-glacial acetic colorimetric approach designed by Huang et al. [48] was used to quantify saponin contents in *A. elata* samples, with oleanolic acid serving as an analytical standard. Briefly, we ground 200 mg of each freeze-dried tissue samples into a fine powder, after which a slightly modified ultrasonic-assisted method [49] was used to fully extract saponins from these samples. Each sample was suspended using a 6 mL volume of 80% ethanol, and the extraction procedure was allowed to proceed for 1 h at 25 °C. Extracts were then filtered before being diluted in a 10 mL volume of 80% ethanol. Next, 100 μL of the filtrate was collected and evaporated in a 70 °C water bath until dry, at which time 400 μL of 5% vanillin-glacial acetic acid and 1.6 mL of perchloric acid were added to the sample, which was then heated for 15 min in a 60 °C water bath, after which it was cooled to 25 °C before 8 mL of ethyl acetate was added. Samples were then mixed thoroughly, and the absorbance at 560 nm (A560) was quantified via a microplate reader (Biotek Elx800, USA). The total saponin content of samples was calculated using the regression eq. Y = 5.31X-0.036 (R^2^ = 0.9995), with Y indicating the A560 and X corresponding to the amount of oleanolic acid (μg).

Ultra-performance liquid chromatography–quadrupole time-of-flight–mass spectrometry (UPLC–QTOF–MS) was used to identify two main sapogenin and three selected araloside monomers that had been isolated previously in *A. elata,* with their retention times and MS data being compared to those of standards to facilitate their identification. For this analysis, a 10 mL volume of 80% methanol was used to ultrasonically extract 100 mg of each freeze-dried tissue sample at 22 °C for 60 min, followed by extract filtration via a 0.22 μm Econofilter. A Waters I Class UPLC–QTOF mass spectrometer (Waters, MA, USA) was used for UPLC–QTOF–MS, with an ACQUITY UPLC BEH C18 analytical column (100 mm × 2.1 mm, 1.7 μm particle size) being used for separation at 40 °C. For this separation, the mobile phase was composed of (A) 0.1% formic acid in water and (B) acetonitrile. The linear gradient conditions were as follows [50]: 0–5.0 min, 5–95% B; 5–11 min, 95% B; 11–12 min, 95–5% B; 12–15 min, 5% B. For each sample, a 5 μl injection volume was used, with a 0.4 mL/min flow rate. The mass spectrometer conducted a full scan in a negative ion mode. N_2_ was used as the desolvation gas. The scanning ranges of the TOF mass and the product ion were 100–2000 m/z and 50–2000 m/z, respectively. Data analysis was performed using the Peakview 2.0/Masterview 1.0 software (AB SCEIX, USA). Five compounds were separated well in 5 min (Additional file 1: Fig. S1), and their molecular formula and retention time (RT) were analyzed using Peakview 2.0/Masterview 1.0 software (Additional file 10: Table S7). For quantitative analyses, each compound was identified repeatedly (*n* = 3), and the height of peaks was used to measure the intensity. Next, standard curves for five standards were prepared and used to calculate saponin contents based on the regression equation (Additional file 10: Table S7). Oleanolic acid, hederagenin, chikusetsusaponin IV, araloside VII and araloside X were from ChemFaces (http://www.chemfaces.cn/) and were used as standards.

### RNA-sequencing

Approximately 100 mg of frozen tissue was then used for total RNA extraction with an OmniPlant RNA Kit based upon provided directions. For RNA-seq analyses, NEBNext, Oligo(dT)25 beads (NEB, USA) were used to specifically enrich for the mRNA present within a 50 μl total RNA sample, after which an NEBNext, Ultra RNA Library Prep Kit for Illumina (NEB) was used to prepare a mRNA library from this enriched samples according to provided directions. An Illumina HiSeq™ 4000 platform was then used for sequencing. The resultant raw reads then underwent quality filtering to remove those reads that were of low quality, contained poly-N sequences, or contained adapter sequences. Clean reads were then de novo assembled with Trinity [51], thereby producing a transcriptomic reference database.

### Functional annotation and pathway analyses

A BLASTx analysis that compared the identified putative unigenes from our transcriptomic database to the nonredundant protein (Nr) database of the National Center for Biotechnology Information (NCBI) (http://www.ncbi.nlm.nih.gov), the Swiss-Prot protein database (http://www.expasy.ch/sprot), and the Clusters of Orthologous Groups (COG)/EuKaryotic Orthologous Groups (KOG) databases (http://www.ncbi.nlm.nih.gov/COG) was next conducted. Furthermore, each unigene was assigned to defined KEGG pathways according to its similarity to genes within the KEGG database (http://www.genome.jp/kegg) as determined via BLAST search, with 1e^− 5^ as the cutoff value. The output of this pathway analysis yielded both enzyme commission (EC) and KEGG orthology (KO) numbers.

### *A. elata* CYP450 and UGT gene identification and phylogenetic analysis

A hidden Markov model (HMM) was retrieved from the Pfam database (http://pfam.sanger.ac.uk) and used for CYP450 and UGT family member identification, with HMMER being used to search the *A. elata* deduced amino acid database for the P450.hmm (PF00067) and UGT.hmm (PF00201) sequences. Those unigenes identified via this initial analysis were then subjected to additional validation with the Simple Modular Architecture Research Tool (SMART; http://smart.embl-heidelberg.de), and open reading frames (ORFs) for these genes were identified using the ORF Finder software (http://bioinf.ibun.unal.edu.co/servicios/sms/orf_find.html). Following the removal of genes encoding protein sequences < 300 amino acids long, 150 CYP450s and 92 UGTs were analyzed further. Multiple sequence alignment was conducted using ClustalX 2.1 software [52]. A neighbor-joining algorithm with a Poisson model and pairwise deletion was used to generate a phylogenetic tree with MEGAX software [53], with 1000 replicates being used for bootstrap testing to validate this tree. EvolView (http://www.evolgenius.info/evolview/) was used for modification of the bootstrap consensus tree, which was exported in the Newick format file [54].

### Assessment of gene expression patterns

The reads per kb per million mapped reads (RPKM) method was used to quantify CYP450 and UGT gene expression levels in the root, stem, and leaf tissues from *A. elata* in this study. TBtools (Toolbox for Biologist, v0.6652) was used for hierarchical clustering analyses. In addition, qRT-PCR was used to validate the RNA-seq results for 23 selected CYP450s and 16 UGTs as follows:

An RNAprep Pure Plant Kit (TianGen, Beijing) was used to isolate RNA from plant tissue samples, after which a ReverTra Ace qPCR RT Master Mix with gDNA Remover (TOYOBO) was used to conduct first-strand cDNA synthesis. A qTOWER real-time PCR system (Analytik Jena, Germany) was then used for qRT-PCR analyses, together with the THUNDERBIRD SYBR qPCR Mix (TOYOBO). As a normalization control, *A. elata GAPDH* was also measured. Thermocycler settings were as follows: 95 °C for 30 s; 40 cycles of 95 °C for 10 s, 55 °C for 10 s, and 72 °C for 15 s. Three biological replicates per sample were analyzed, and the 2^−△△CT^ method was used to quantify gene expression results. Primers used in this study are compiled in Additional file 11: Table S8.

### Subcellular localization analysis

We selected the CYP716A295, CYP716A296, and CYP72A763 genes to assess representative CYP450 subcellular localization by PCR-amplifying the ORFs for these genes without a stop codon using specific primers with corresponding enzyme sites (Additional file 11: Table S8). Sangon Biotech (Shanghai, China) then conducted sequence validation of the isolated PCR products, after which they were inserted upstream of enhanced green fluorescent protein (GFP) at an appropriate restriction enzyme digestion site in the pAN580-35S-GFP vector, yielding pAN580-35S-CYP450::GFP vectors. These recombinant plasmids were transformed into *Arabidopsis* protoplasts along with the DEP2-RFP plasmid using a polyethylene glycol (PEG)-mediated transient transformation system [55]. Protoplasts expressing the resultant GFP fusion proteins were then visualized via Airyscan confocal laser scanning microscope (ZEISS710, Carl Zeiss, Jena, Germany).

## Supplementary information

**Additional file 1: Fig. S1.** Typical ion current (TIC) chromatograms for aralosides in leaves (A), stems (B) and roots (C) of *A. elata* and of the mixed reference substance (D) as identified via UPLC–QTOF–MS. Peak numbers correspond to these different aralosides, including: araloside VII (1), araloside X (2), chikusetsusaponin IV (3), hederagenin (4) and oleanolic acid (5).

**Additional file 2: Fig. S2.** Venn diagram indicating annotated genes by the KEGG, KOG, Nr and Swissprot databases. The number of genes annotated is listed in each diagram section.

**Additional file 3: Table S1.** Unigenes related to saponin skeleton biosynthesis obtained after three independent biological replicates along with their mean values.

**Additional file 4: Table S2.** List of 150 CYP450s of *A. elata* identified in this study.

**Additional file 5: Table S3.** List of 92 UGTs of *A. elata* identified in this study.

**Additional file 6: Table S4.** Functionally characterized UGTs from *Arabidopsis* and other plant species.

**Additional file 7: Fig. S3.** qRT-PCR was used to validate the expression levels of randomly selected CYP450s from our RNA-seq study.

**Additional file 8: Table S5.** A list of 36 previously reported plant CYP450s involved in triterpenoid biosynthesis.

**Additional file 9: Table S6.** List of 16 previously reported plant UGTs that play roles in triterpenoid biosynthesis.

**Additional file 10: Table S7.** List of five standards analyzed by UPLC- QTOF- MS.

**Additional file 11: Table S8.** Sequences of the primers used in this study.

## Data Availability

All RNA-seq reads generated by this study are publicly available at the NCBI Short Read Archive (SRA) under accession number SRP216867 (https://trace.ncbi.nlm.nih.gov/Traces/sra/?study=SRP216867) and BioProject accession PRJNA555256 (https://www.ncbi.nlm.nih.gov/Traces/study/?acc=PRJNA555256).

## Abbreviations

*A. elata*: *Aralia elata* (Miq.) Seem
RNA-seq: RNA-sequencing
CYP450: Cytochrome P450
qRT-PCR: Quantitative real-time reverse transcription PCR
SRSs: Substrate recognition sites
ER: Endoplasmic reticulum
IPP: Isopentenyl diphosphate
DMAPP: Dimethylallyl pyrophosphate
MVA: Mevalonate
MEP: Methylerythritol 4-phosphate
OSC: Oxidosqualene cyclase
UGTs: UDP-glycosyltransferases
AACT: Acetyl-CoA acetyltransferase
HMGS: Hydroxymethyl glutaryl CoA synthase
HMGR: Hydroxymethyl gutryl CoA reductase
MVK: Mevalonate kinase
PMK: Phosphomevalonate kinase
MVD: Diphosphosphate decarboxylase
IDI: Isopentenyl pyrophosphate
DXS: 1-deoxy-D-xylulose-5-phosphate synthase
DXR: 1-deoxy-D-xylulose-5-phosphate reductoisomerase
MEP-CT: 2-C-methyl-D-erythritol 4-phosphate cytidylyltransferase
CDP-MEK: 4-diphosphocytidyl-2-C-methyl-D-erythritol kinase
MECDPS: 2-C-methyl-D-erythritol 2,4-cyclodiphosphate synthase
HMBPPS: (E)-4-hydroxy-3-methylbut-2-enyl-diphosphate synthase
HMBPPR: 4-hydroxy-3-methylbut-2-en-1-yl diphosphate reductase
GPS: Geranyl diphosphate synthase
FPS: Farnesyl diphosphate synthase
SS: Squalene synthase
SE: Squalene monooxygenase
bAS: β-amyrin synthase
