# Supplementary figures and images for: Identification and analysis of CYP450 and UGT supergene family members from the transcriptome of Aralia elata (Miq.) seem reveal candidate genes for triterpenoid saponin biosynthesis
Source: BMC Plant Biol. 2020 May 13;20:214. doi: 10.1186/s12870-020-02411-6 (PMC7218531; doi:10.1186/s12870-020-02411-6)

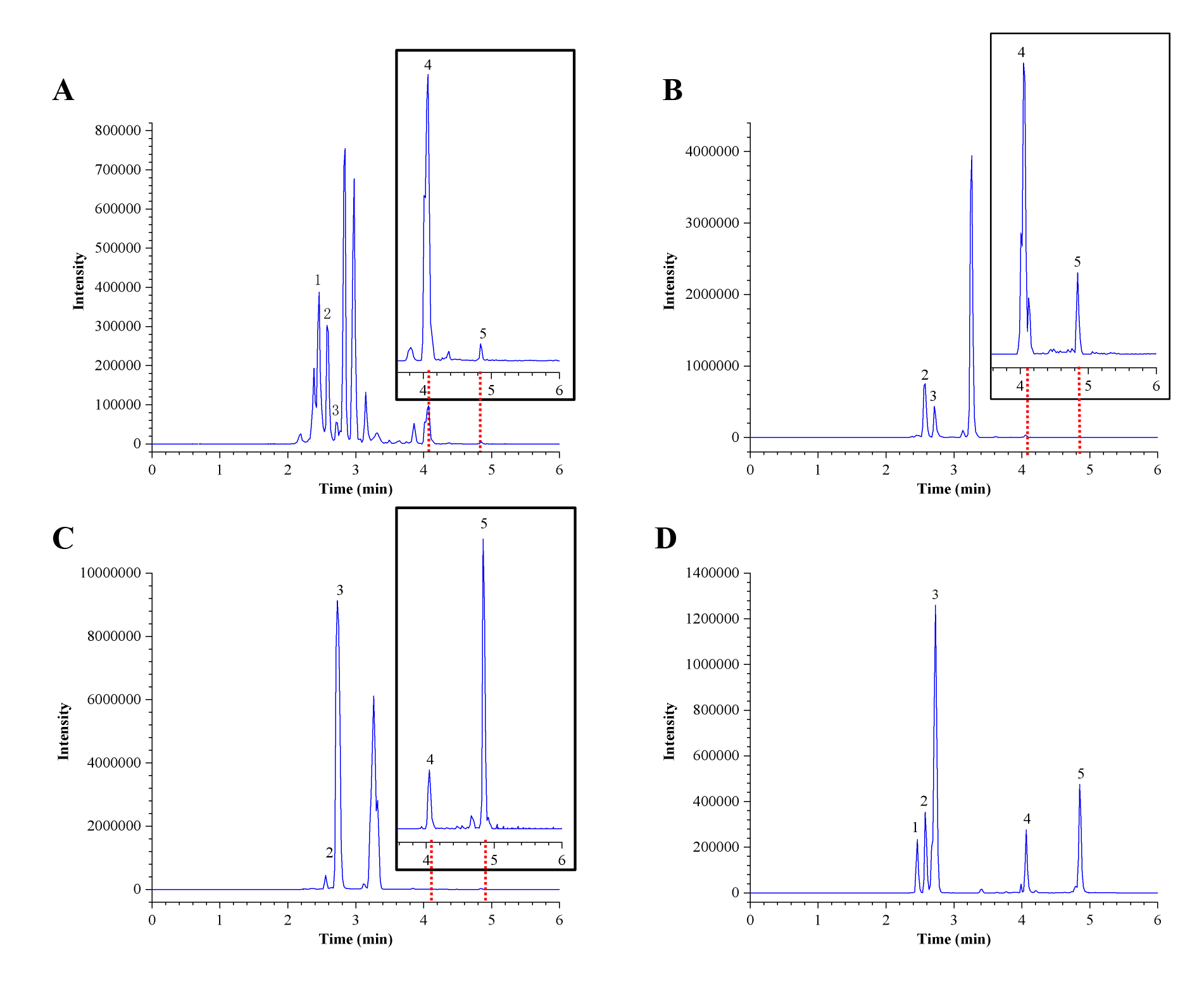

Supplement: Supplementary file 1 — Additional file 1: Fig. S1. Typical ion current (TIC) chromatograms for aralosides in leaves (A), stems (B) and roots (C) of A. elata and of the mixed reference substance (D) as identified via UPLC–QTOF–MS. Peak numbers correspond to these different aralosides, including: araloside VII (1), araloside X (2), chikusetsusaponin IV (3), hederagenin (4) and oleanolic acid (5). [file 12870_2020_2411_MOESM1_ESM.tif]

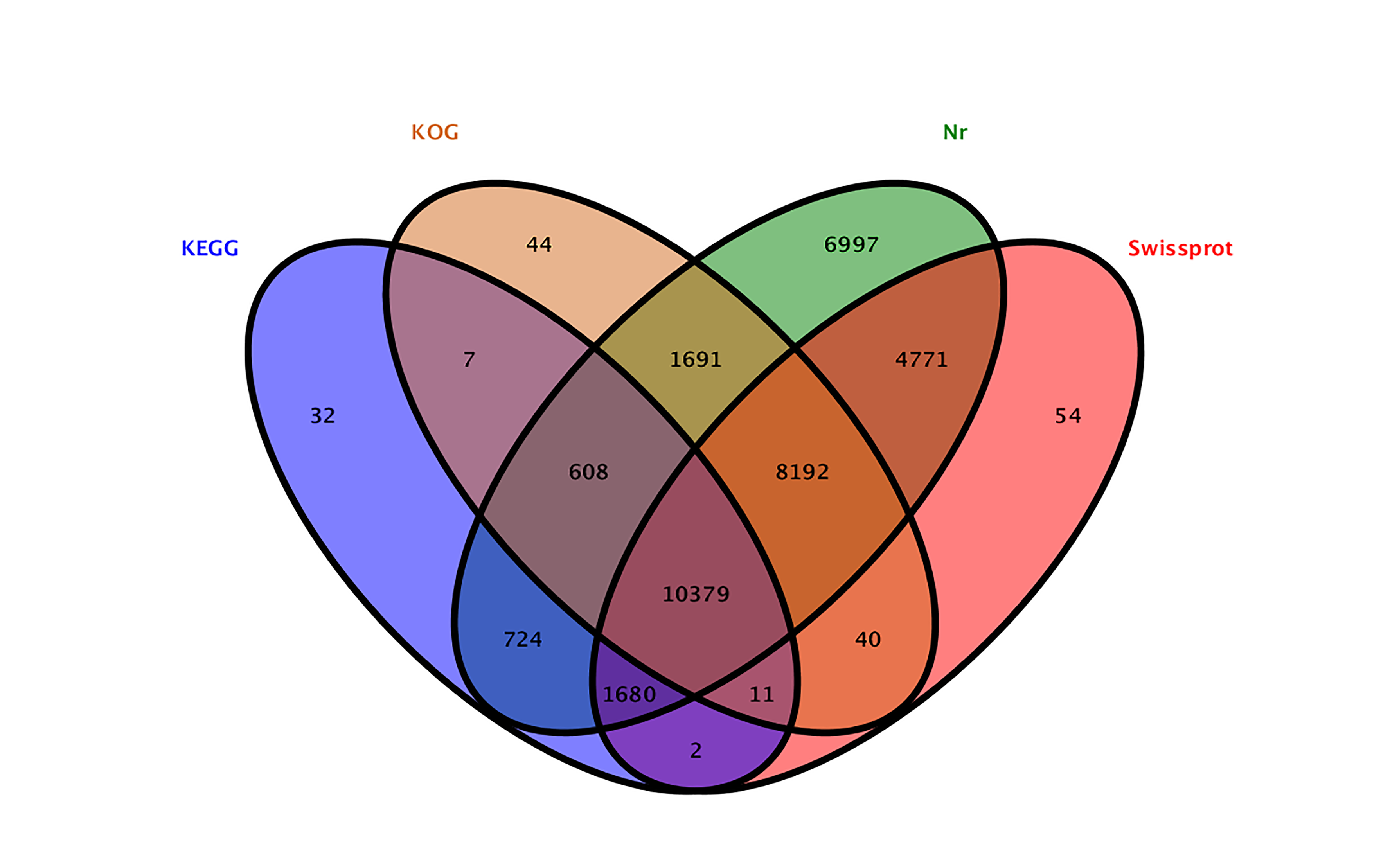

Supplement: Supplementary file 2 — Additional file 2: Fig. S2. Venn diagram indicating annotated genes by the KEGG, KOG, Nr and Swissprot databases. The number of genes annotated is listed in each diagram section. [file 12870_2020_2411_MOESM2_ESM.tif]

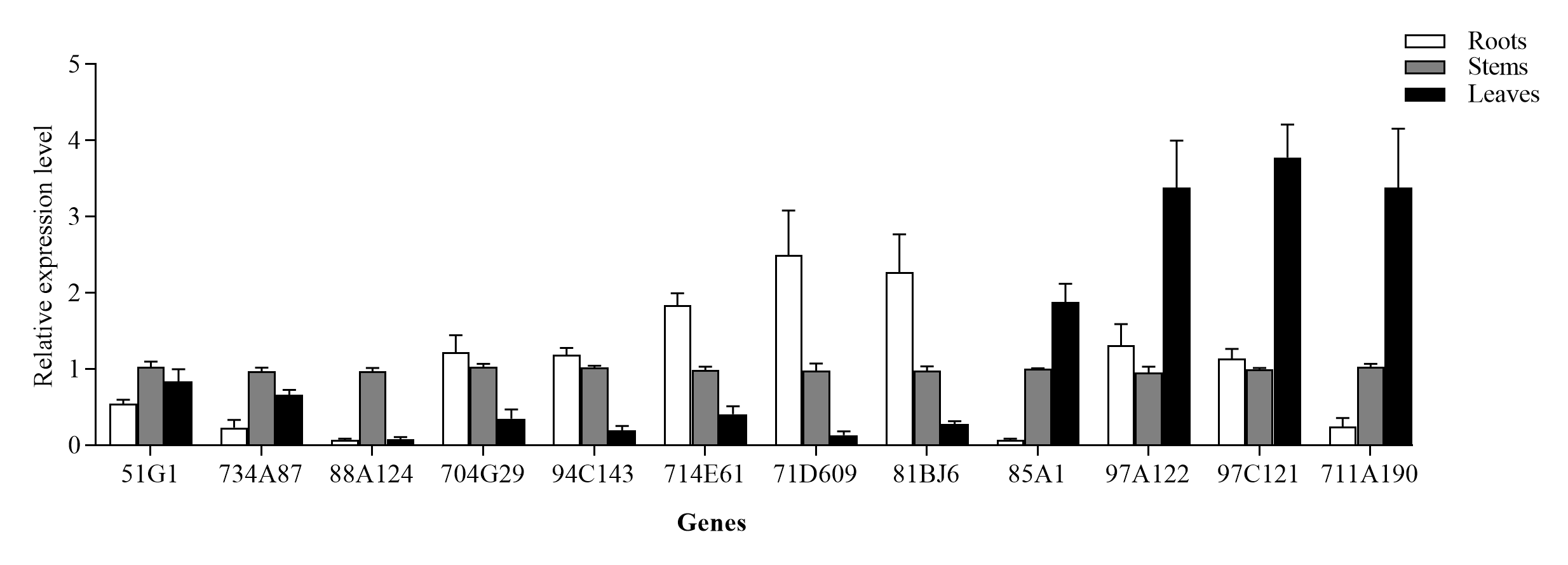

Supplement: Supplementary file 7 — Additional file 7: Fig. S3. qRT-PCR was used to validate the expression levels of randomly selected CYP450s from our RNA-seq study. [file 12870_2020_2411_MOESM7_ESM.tif]
