# Supplementary material for: Identification and analysis of CYP450 and UGT supergene family members from the transcriptome of Aralia elata (Miq.) seem reveal candidate genes for triterpenoid saponin biosynthesis
Source: BMC Plant Biol. 2020 May 13;20:214. doi: 10.1186/s12870-020-02411-6 (PMC7218531; doi:10.1186/s12870-020-02411-6)
Supplement: Supplementary file 3 — Additional file 3: Table S1. Unigenes related to saponin skeleton biosynthesis obtained after three independent biological replicates along with their mean values. [file 12870_2020_2411_MOESM3_ESM.docx]

**Table S1** Unigenes related to saponin skeleton biosynthesis obtained after three independent biological replicates along with their mean values.

| **Definition** | **Abbreviation** | **EC number** | **KO assignment** | **Number of unigene** | **RPKM** | | | | |
| --- | --- | --- | --- | --- | --- | --- | --- | --- | --- |
|  |  |  |  |  | **Root** | | **Stem** | | **Leaf** |
| Acetyl-CoA acetyltransferase | AACT | 2.3.1.9 | K00626 | 2 | 15.84 | 17.08 | | 23.52 | |
| Hydroxymethyl glutaryl CoA synthase | HMGS | 2.3.3.10 | K01641 | 3 | 221.75 | 97.63 | | 132.32 | |
| Hydroxymethyl gutryl CoA reductase | HMGR | 1.1.1.34 | K00021 | 5 | 65.35 | 44.23 | | 155.89 | |
| Mevalonate kinase | MVK | 2.7.1.36 | K00869 | 1 | 23.10 | 16.70 | | 9.78 | |
| Phosphomevalonate kinase | PMK | 2.7.4.2 | K00938 | 1 | 6.37 | 7.47 | | 7.46 | |
| Diphosphosphate decarboxylase | MVD | 4.1.1.33 | K01597 | 2 | 77.61 | 56.83 | | 82.77 | |
| Isopentenyl pyrophosphate | IDI | 5.3.3.2 | K01823 | 2 | 60.12 | 71.54 | | 109.80 | |
| 1-deoxy-D-xylulose-5-phosphate synthase | DXS | 2.2.1.7 | [K01662](http://www.genome.jp/dbget-bin/www_bget?ko:K01662) | 6 | 59.1 | 37.93 | | 202.45 | |
| 1-deoxy-D-xylulose-5-phosphate reductoisomerase | DXR | 1.1.1.267 | K00099 | 2 | 32.47 | 32.40 | | 83.07 | |
| 2-C-methyl-D-erythritol 4-phosphate cytidylyltransferase | MEP-CT | 2.7.7.60 | K00991 | 2 | 3.07 | 3.04 | | 13.67 | |
| 4-diphosphocytidyl-2-C-methyl-D-erythritol kinase | CDP-MEK | 2.7.1.148 | K00919 | 1 | 51.44 | 40.69 | | 88.36 | |
| 2-C-methyl-D-erythritol 2,4-cyclodiphosphate synthase | MECDPS | 4.6.1.12 | K01770 | 3 | 10.22 | 21.11 | | 116.55 | |
| (E)-4-hydroxy-3-methylbut-2-enyl-diphosphate synthase | HMBPPS | 1.17.7.1 | K03526 | 4 | 93.95 | 81.99 | | 203.64 | |
| 4-hydroxy-3-methylbut-2-en-1-yl diphosphate reductase | HMBPPR | 1.17.1.2 | K03527 | 1 | 40.91 | 39.29 | | 97.87 | |
| Geranyl diphosphate synthase | GPS | 2.5.1.29 | K14066 | 3 | 23.61 | 20.70 | | 78.77 | |
| Farnesyl diphosphate synthase | FPS | 2.5.1.10 | K00787 | 5 | 39.50 | 54.22 | | 91.58 | |
| Squalene synthase | SS | 2.5.1.21 | K00801 | 2 | 123.13 | 129.64 | | 75.55 | |
| Squalene monooxygenase | SE | 1.14.14.17 | [K00511](http://www.genome.jp/dbget-bin/www_bget?ko:K00511target=_blank) | 8 | 14.43 | 36.36 | | 78.45 | |
| β-amyrin synthase | bAS | 5.4.99.39 | [K15813](http://www.genome.jp/dbget-bin/www_bget?ko:K15813) | 13 | 30.14 | 52.65 | | 88.46 | |
