# Supplementary material for: Identification and analysis of CYP450 and UGT supergene family members from the transcriptome of Aralia elata (Miq.) seem reveal candidate genes for triterpenoid saponin biosynthesis
Source: BMC Plant Biol. 2020 May 13;20:214. doi: 10.1186/s12870-020-02411-6 (PMC7218531; doi:10.1186/s12870-020-02411-6)
Supplement: Supplementary file 6 — Additional file 6: Table S4. Functionally characterized UGTs from Arabidopsis and other plant species. [file 12870_2020_2411_MOESM6_ESM.docx]

**Table S4** UGTs from *Arabidopsis thaliana* and other plant species.

| **Genes** | **Species** | **Accession number** | **Group** |
| --- | --- | --- | --- |
| AtUGT73C5 | *Arabidopsis thaliana* | OAP09184 | D |
| AtUGT73C6 | *Arabidopsis thaliana* | OAP07438 | D |
| AtUGT85A1 | *Arabidopsis thaliana* | NP_173656.1 | G |
| AtUGT74B1 | *Arabidopsis thaliana* | NP_173820.1 | L |
| AtUGT89B1 | *Arabidopsis thaliana* | NP_177529.2 | B |
| AtUGT90A1 | *Arabidopsis thaliana* | NP_179281.3 | C |
| AtUGT71C1 | *Arabidopsis thaliana* | NP_180536.1 | E |
| AtUGT87A1 | *Arabidopsis thaliana* | NP_180576.1 | J |
| AtUGT86A1 | *Arabidopsis thaliana* | NP_181234.1 | K |
| AtUGT83A1 | *Arabidopsis thaliana* | NP_186859.1 | I |
| AtUGT76B1 | *Arabidopsis thaliana* | NP_187742.1 | H |
| AtUGT82A1 | *Arabidopsis thaliana* | NP_188864.1 | N |
| AtUGT84A1 | *Arabidopsis thaliana* | NP_193283.2 | L |
| AtUGT72C1 | *Arabidopsis thaliana* | NP_195395.4 | E |
| AtUGT92A1 | *Arabidopsis thaliana* | NP_196793.1 | M |
| AtUGT79B1 | *Arabidopsis thaliana* | NP_200217.1 | A |
| AtUGT75B1 | *Arabidopsis thaliana* | NP_563742.1 | L |
| AtUGT78D1 | *Arabidopsis thaliana* | NP_564357.1 | F |
| AtUGT91A1 | *Arabidopsis thaliana* | NP_565540.4 | A |
| AtUGT88A1 | *Arabidopsis thaliana* | NP_566550.1 | E |
| AtUGT73B1 | *Arabidopsis thaliana* | NP_567955.1 | D |
| CaUGT95B1 | *Cicer arietinum* | AGU14123.1 | M |
| CsUGT84A22 | *Crocus sativus* | ALO19890 | L |
| CsUGT78A14 | *Crocus sativus* | ALO19888 | F |
| MtUGT71G1 | *Medicago truncatula* | AAW56092 | E |
| OsUGT706C1 | *Oryza sativa* | BAB68090 | E |
| OsUGT706D1 | *Oryza sativa* | BAB68093 | E |
| OsUGT709A4 | *Oryza sativa* | BAC80066 | P |
| PgUGT94Q2 | *Panax ginseng* | AGR44632 | A |
| PgUGT74AE2 | *Panax ginseng* | AGR44631 | L |
| GRMZM2G110511/P01 | *Zea mays* | AY082660 | O |
| GRMZM5g834303/P01 | *Zea mays* | NM_001155519.3 | P |
| GRMZM2G074395/P01 | *Zea mays* | NM_001156290.2 | Q |
