# Supplementary material for: Identification and analysis of CYP450 and UGT supergene family members from the transcriptome of Aralia elata (Miq.) seem reveal candidate genes for triterpenoid saponin biosynthesis
Source: BMC Plant Biol. 2020 May 13;20:214. doi: 10.1186/s12870-020-02411-6 (PMC7218531; doi:10.1186/s12870-020-02411-6)
Supplement: Supplementary file 8 — Additional file 8: Table S5. A list of 36 previously reported plant CYP450s involved in triterpenoid biosynthesis. [file 12870_2020_2411_MOESM8_ESM.docx]

**Table S5** A list of 36 previously reported plant CYP450s involved in triterpenoid biosynthesis.

| **Gene name** | **Species** | **Accession No.** | **Enzymatic activity** | **Sapogenin** |
| --- | --- | --- | --- | --- |
| CYP51H10 | *Avena strigosa* | ABG88961 | C-12, 13β epoxidase; C-16β oxidase | Oleanane |
| CYP71A16 | *Arabidopsis thaliana* | NP_199073 | C-23 oxidase | Marnerol, Marneral, |
| CYP71D353 | *Lotus japonicus* | AHB62239 | C-20, C-28 oxidase | Lupane |
| CYP72A61v2 | *Medicago truncatula* | BAL45199 | C-23 oxidase | Oleanane |
| CYP72A63 | *Medicago truncatula* | BAL45200 | C-30 oxidase | Oleanane |
| CYP72A67 | *Medicago truncatula* | ABC59075 | C-2β oxidase | Oleanane |
| CYP72A68v2 | *Medicago truncatula* | BAL45204 | C-23 oxidase | Oleanane |
| CYP72A154 | *Glycyrrhiza uralensis* | AB558153 | C-30 oxidase | Oleanane |
| CYP87D16 | *Maesa lanceolata* | AHF22090 | C-16α oxidase | Oleanane |
| CYP88D6 | *Glycyrrhiza uralensis* | AB433179 | C-11 oxidase | Oleanane |
| CYP93E1 | *Glycine max* | BAE94181 | C-24 oxidase | Oleanane |
| CYP93E2 | *Medicago truncatula* | ABC59085 | C-24 oxidase | Oleanane |
| CYP93E3 | *Glycyrrhiza uralensis* | BAG68930 | C-24 oxidase | Oleanane |
| CYP705A1 | *Arabidopsis thaliana* | NP_193268 | C-15, C-16 cleavarage | Arabidiol |
| CYP708A2 | *Arabidopsis thaliana* | NP_851153 | C-7β oxidase | Thaianol |
| CYP716A1 | *Arabidopsis thaliana* | NP_198460 | C-28 oxidase | Oleanane,ursane,Lupane |
| CYP716A2 | *Arabidopsis thaliana* | BAU61505 | C-28, C-22α oxidase | Oleanane |
| CYP716A14v2 | *Artemisia annua* | AHF22083 | C-3 oxidase | Ursane |
| CYP716A12 | *Medicago truncatula* | ABC59076 | C-28 oxidase | Oleanane,ursane,Lupane |
| CYP716A15 | *Vitis vinifera* | BAJ84106 | C-28 oxidase | Oleanane,ursane,Lupane |
| CYP716A17 | *Vitis vinifera* | BAJ84107 | C-28 oxidase | Oleanane |
| CYP716A47 | *Panax ginseng* | AEY75212 | C-12 oxidase | Dammarane |
| CYP716A52v2 | *Panax ginseng* | AFO63032 | C-28 oxidase | Oleanane |
| CYP716A53v2 | *Panax ginseng* | AFO63031 | C-6 oxidase | Dammarane |
| CYP716A75 | *Maesa lanceolata* | AHF22088 | C-28 oxidase | Oleanane |
| CYP716A78 | *Chenopodium quinoa* | ANY30853 | C-28 oxidase | Oleanane |
| CYP716A79 | *Chenopodium quinoa* | ANY30854 | C-28 oxidase | Oleanane |
| CYP716A80 | *Barbarea vulgaris subsp. Arcuata* | ALR73782 | C-28 oxidase | Oleanane |
| CYP716A81 | *Barbarea vulgaris subsp. Arcuata* | ALR73781 | C-28 oxidase | Oleanane |
| CYP716A179 | *Glycyrrhiza uralensis* | BAW34647 | C-28 oxidase | Oleanane,ursane,Lupane |
| CYP716AL1 | *Catharanthus roseus* | AEX07773 | C-28 oxidase | Oleanane,ursane,Lupane |
| CYP716Y1 | *Bupleurum falcatum* | AHF45909 | C-16α oxidase | Oleanane |
| CYP716A141 | *Platycodon grandiflorus* | LC209200 | C-16β oxidase | Oleanane |
| CYP716A140v2 | *Platycodon grandiflorus* | LC209199 | C-28 oxidase | Oleanane |
| CYP716A94  CYP72A397 | *Kalopanax septemlobus*  *Kalopanax septemlobus* | ALO23113  ALO23117.1 | C-28 oxidase  C-23 oxidase | Oleanane  Hederagenin |
