# Supplementary material for: Identification and analysis of CYP450 and UGT supergene family members from the transcriptome of Aralia elata (Miq.) seem reveal candidate genes for triterpenoid saponin biosynthesis
Source: BMC Plant Biol. 2020 May 13;20:214. doi: 10.1186/s12870-020-02411-6 (PMC7218531; doi:10.1186/s12870-020-02411-6)
Supplement: Supplementary file 9 — Additional file 9: Table S6. List of 16 previously reported plant UGTs that play roles in triterpenoid biosynthesis. [file 12870_2020_2411_MOESM9_ESM.docx]

**Table S6** A list of 16 previously reported plant UGTs involved in triterpenoid biosynthesis

| **Gene name** | **Species** | **Accession No.** | **Enzymatic activity** |
| --- | --- | --- | --- |
| UGT71A27 | *Panax ginseng* | KM491309 | Protopanaxadiol 20-O-glucosyltransferase |
| UGT74AE2 | *Panax ginseng* | AGR44631 | Protopanaxadiol and compund K 3-O-glucosyltransferase |
| UGT94Q2 | *Panax ginseng* | AGR44632 | Ginsenoside Rh_2_ and F_2_ 3-O-glucosyltransferase |
| OAGT1 | *Panax zingiberensis* | MH819284 | Oleanolic acid 3-O-glucuronosyltransferase |
| OAGT2 | *Panax zingiberensis* | MH819285 | Oleanolic acid 3-O-glucuronosyltransferase |
| OAGT3 | *Panax zingiberensis* | MH819286 | Oleanolic acid 3-O-glucuronosyltransferase |
| OAGT | *Panax japonicus var.major* | MH819287 | Oleanolic acid 3-O-glucuronosyltransferase |
| UGT71G1 | *Medicago truncatula* | AAW56092 | Medicagenic acid glucosyltransferase |
| UGT73K1 | *Medicago truncatula* | AAW56091 | Hederagenin and soyasapogenols B and E glucosyltransferase |
| UGT73F3 | *Medicago truncatula* | ACT34898 | Hederagenin 28-O-glucosyltransferase |
| UGT74M1 | *Saponaria vaccaria* | ABK76266 | Gypsogenic acid 28-O-glucosyltransferase |
| UGT73P2 | *Glycine max* | BAI99584 | Soyasapogenol B 3-O-galactosyltransferase |
| UGT91H4 | *Glycine max* | BAI99585 | Soyasapogenol III 3-O-rhamnosyltransferase |
| UGT73F4 | *Glycine max* | BAM29363 | Saponin A0-αg 22-O-xylosyltransferase |
| UGT73F2 | *Glycine max* | BAM29362 | Saponin A0-αg 22-O-glucosyltransferase |
| UGT73C10 | *Barbarea vulgaris* | AFN26666 | Oleanolic acid or hederagenin 3-O-glucosyltransferase |
| UGT73C11 | *Barbarea vulgaris* | AFN26667 | Oleanolic acid or hederagenin 3-O-glucosyltransferase |
| UGT73C12 | *Barbarea vulgaris* | AFN26668 | Oleanolic acid or hederagenin 3-O-glucosyltransferase |
| UGT73C13 | *Barbarea vulgaris* | AFN26669 | Oleanolic acid or hederagenin 3-O-glucosyltransferase |
| GuUGAT | *Glycyrrhiza uralensis* | KT759000 | Glycyrrhetinic acid 3-O-glucuronosyltransferase |
