# Supplementary material for: Identification and analysis of CYP450 and UGT supergene family members from the transcriptome of Aralia elata (Miq.) seem reveal candidate genes for triterpenoid saponin biosynthesis
Source: BMC Plant Biol. 2020 May 13;20:214. doi: 10.1186/s12870-020-02411-6 (PMC7218531; doi:10.1186/s12870-020-02411-6)
Supplement: Supplementary file 10 — Additional file 10: Table S7. List of five standards analyzed by UPLC- QTOF- MS. [file 12870_2020_2411_MOESM10_ESM.docx]

**Table S7** List of five standards analyzed by UPLC–QTOF–MS.

| **Standards** | **RT**  **(min)** | **Molecular**  **Formula** | **Expected m/z** | **Found at m/z** | **Error (ppm)** | **Equation of**  **linear regression** | **Correlation (R^2^)** |
| --- | --- | --- | --- | --- | --- | --- | --- |
| Oleanolic acid | 4.86 | C30H48O3 | 455.3531 | 455.3529 | -0.4 | Y=120517X+26438 | 0.9921 |
| Hederagenin | 4.04 | C30H48O4 | 471.3480 | 471.3477 | -0.7 | Y=33473X-4102.2 | 0.9895 |
| Chikusetsusaponin IV | 2.73 | C47H74O18 | 927.4948 | 927.4968 | 2.1 | Y=2*10^6X-451606 | 0.9923 |
| Araloside VII | 2.46 | C54H88O24 | 1119.5593 | 1119.5617 | 2.2 | Y=559605X-303670 | 0.9948 |
| Araloside X | 2.58 | C60H98O28 | 1266.6245 | 1266.6232 | -1.4 | Y=258238X-74672 | 0.9922 |
